# Fatty Acid Synthase Beta Dehydratase in the Lipid Biosynthesis Pathway Is Required for Conidiogenesis, Pigmentation and Appressorium Formation in *Magnaporthe oryzae* S6

**DOI:** 10.3390/ijms21197224

**Published:** 2020-09-30

**Authors:** Vaanee Sangappillai, Kalaivani Nadarajah

**Affiliations:** Department of Biological Sciences and Biotechnology, Faculty of Science and Technology, Universiti Kebangsaan Malaysia; UKM Bangi 43600, Malaysia; s.vaanee88@hotmail.com

**Keywords:** *Magnaporthe oryzae*, lipid biosynthesis, appressorium, fatty acid, peroxisomes

## Abstract

Lipid biosynthesis produces glycerol, which is important in fueling turgor pressure necessary for germination and penetration of plant host by fungi. As the relationship between pathogenicity and the lipid biosynthetic pathway is not fully understood, we have elucidated the role of the fatty acid synthase beta subunit dehydratase (*FAS1*) gene in lipid biosynthesis. The *FAS1* gene was silenced through homologous double crossover in *Magnaporthe oryzae* strain S6 to study the effect on lipid biosynthesis. The vegetative growth of *Δfas1* mutants show the highest drop on oleic acid (between 10 and 50%), while the mycelial dry weight of mutants dropped significantly on all media. Conidiation of FAS1 mutants show a ~10- and ~5-fold reduction on oatmeal and Potato Dextrose Agar (PDA), respectively. Mutants formed mycelium that were mildly pigmented, indicating that the deletion of *FAS1* may have affected melanin biosynthesis. Biochemical and gene expression studies concluded that the fatty acid degradation pathway might have been interrupted by *FAS1* deletion. FAS1 mutants showed no enzyme activity on glucose or olive oil, suggesting that the mutants may lack functional peroxisomes and be defective in β-oxidation of fatty acids, hence explaining the reduced lipid deposits in the spores.

## 1. Introduction

Asia has the largest rice (*Oryza sativa* L.) growing area, which includes countries such as China, India, Thailand and Vietnam [1]. As in all agricultural crops, rice is susceptible to biotic and abiotic stresses. The major biotic factors affecting rice farmers in Malaysia are bacterial leaf blight, rice blast and sheath blight. The rice blast disease of cultivated rice is a problem compounded by climate change and modern agricultural practices that makes this disease uncontrollable, resulting in major yield losses worldwide [2,3,4]. This disease threatens global food security, where it is estimated that enough rice to feed 60 million people is destroyed by rice blast disease yearly [5,6,7,8]. The causative agent of this disease is an ascomycete fungus, *Magnaporthe oryzae* (synonym of *Pyricularia oryzae*).

The penetration process of this fungus into the plant’s cuticle is initiated when the three-celled, teardrop-shaped conidia lands on the hydrophobic surface of rice leaves and initiates the rice blast symptoms. The germ tube extends and differentiates into bulbous melanized, specialized dome-shaped structures called appressorium, which contains cell wall-degrading enzymes and melanin-rich cell walls [9,10,11,12]. Conidiogenesis is a process that involves a series of morphological events that leads to the growth of hypha and eventually, conidiation. The morphology of the conidia and mycelium reflects on the pathogenicity of the fungus [13].

*M. oryzae* spores contain storage reserves in the form of trehalose, glycogen and lipids which are mobilized when the germ tube is formed. The glycerol produced via lipid biosynthesis fuels turgor pressure necessary for the penetration of the appressorium. Lipid is essential for the virulence of *Magnaporthe,* as the entire plant infection process, from spore germination to development of the penetration hypha, is fueled by storage reserves carried in the spores [14]. High levels of triacylglycerol lipase and glycerol have been measured in appressoria during turgor pressure [9,14] It has been shown that pressure of up to 8.0 MPa is generated by the appressorium at the point of penetration [15,16]. The glycerol acts as a highly soluble osmolyte, causing rapid influx of water generating hydrostatic turgor pressure [10]. Vacuoles in *M. oryzae* are lipid storage reserves that are degraded to serve as energy or osmotically activate metabolites such as glycerol, for turgor pressure during penetration [17]. 

The appressorium is rich in chitin and contains a layer of darkly pigmented melanin on the inside of the cell wall. The melanin layer is important for the fungus to withstand the physical force produced during host penetration [15,16]. Besides, the melanin layer maintains cell wall rigidity in the appressoria, which is essential to focus the turgor pressure during vertical penetration [9,18]. *M. oryzae* produces melanin via polyketide biosynthesis and is responsible for the formation of melanized colonies. The site of melanin deposits in this fungus is essential for host penetration [9]. The fungus enters the underlying epidermal cell to form biotrophic invasive hyphae [12,14]. Here, the fungus develops bulbous, branched invasive hyphae and lobed infection hyphae which are bound by the invaginated plasmalemma in and between the plant cell [12]. 

**Figure 1:**
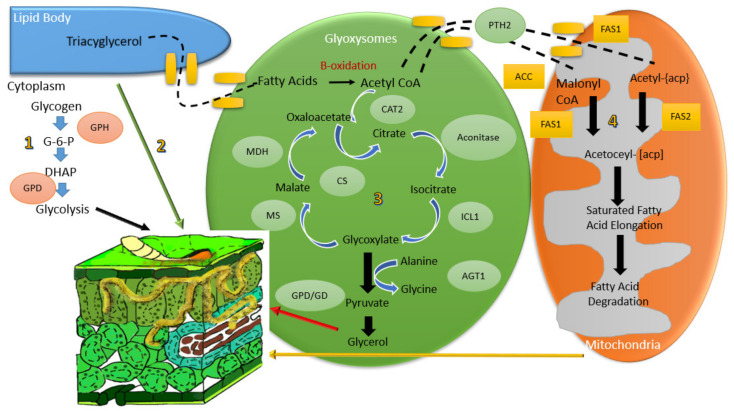
Model of turgor pressure generation involving fatty acids, glycogen, glycerol and triacylglycerols in *M. oryzae.* Pathway (**1**) is where glycogen is carried into the glycolysis pathway to produced pyruvate, which then enters Krebs cycle for energy production for penetration of fungus into host [25]. Pathway (**2**) involves lipid bodies moving into the appressorium during maturation, where they are degraded by triacylglycerol lipase during turgor generation [26]. Pathway (**3**) explains the glycoxylate cycle that produces glycerol. Pathway (**4**) involves the fatty acid synthesis at the top of the route and ends with fatty acid degradation for turgor pressure of the appressorium [23].

Fatty acid, glycogen, glycerol and triacylglycerol pathways are believed to contribute towards appressorium turgor pressure in *M. oryzae,* as described in Figure 1 [14]. The fatty acid β-oxidation occurs in peroxisomes and contributes to the generation of acetyl-CoA, which leads to melanin and lipid biosynthesis, as well as the activation of the glyoxylate and gluconeogenesis cycles during plant infection [14]. Lipid biosynthesis (lipogenesis) takes place in the cytoplasm where acetyl-CoA is used as the precursor [14]. Lipid bodies are transported into appressorial vacuoles during appressorium formation where rapid lipolysis is stimulated. Lipid bodies are then broken down into fatty acids by triacylglycerol lipases that consequently generate acetyl-CoA and glycerol [14]. Fatty acids are processed by β-oxidation and the mechanism involves four major enzymes in the reaction. Through these four steps, a two-carbon unit is split into acetyl-CoA that is used to produce glycerol by way of glyoxylate and gluconeogenesis cycles [12]. Glycogen degradation that is regulated by glycogen phosphorylase (GPH1) releases glucose in the form of glucose-1-phosphate within the appressorium during turgor pressure. 

Many genes in fatty acid catabolism have been studied in *Magnaporthe* sp. For instance, deletion studies of fatty acyl-CoA reductase 1 (*FAR1)* and fatty acyl-CoA reductase 2 *(FAR2)* in *M. oryzae* result in a phenotype consistent with fatty acid utilization and demonstrates involvement in gene function associated with lipolysis, fatty acid β-oxidation, peroxisome function and gluconeogenesis [16]. Further, Deng et al. showed that *MoPEX1* plays an essential role in peroxisomal function for infection-related morphogenesis through degradation of lipid droplets and mobilization through appressorium development in *M. grisea* [19]. Mutants of alanine glyoxylate aminotransferase 1 (*AGT1*) [20], peroxisomal carnitine acetyl transferase (*CAT2)* gene [12], and mitogen-activated protein kinase (PMK1 MAPK) [21] in *Magnaporthe* sp. showed β-oxidation, and mobilization of lipid bodies to the germ tube and appressorium that affects the infection process. Further, the *MPG1*-hydrophobin encoding gene was shown to be important in the elongation of the germ tube and attachment to the hydrophobic leaf surface in *M. oryzae* [22]. *PTH11* is involved in the upstream signaling of cyclic adenosine monophosphate (cAMP)and appressorium differentiation [18]. Previous research has demonstrated that degradation of lipid and glycogen reserves by catalytic subunit of protein kinase (CPKA)/ Transcription factor SUM-1 (*SUM1*) encodes protein kinase A (PKA) holoenzymes, which contributes towards turgor pressure [23]. Glycerol generation is achieved by degradation of triacylglycerol by triacylglycerol lipase, which is regulated by the CPKA-encoded PKA [21].

Following a proteomic study conducted on *M. oryzae* S6 in our laboratory [24], we identified potential key players in the lipid biosynthetic pathway. Genes that were directly correlated to virulence and pathogenicity were shortlisted. We proceeded to conduct a functional analysis of fatty acid synthase beta subunit dehydratase (*FAS1*), an upstream gene in lipid biosynthesis from kyoto encyclopedia of genes and genomes (KEGG). Though the role of *FAS1* has been studied in bacteria, its role in fungi, specifically *M. oryzae,* has not been elucidated. Through homologous double crossover, mutants were generated and the effect of *FAS1* mutation on conidiogenesis, pigmentation, lipid biosynthesis and appressorium formation of *M. oryzae* was studied. 

## 2. Results and Discussion

### 2.1. Identification of FAS1 in M. Oryzae Strain S6

Lipid biosynthesis and glycerol degradation are factors proven to contribute to appressorium maturation and turgor generation during pathogenicity [23]. The lipid biosynthetic pathway of this fungus was analyzed in KEGG, and several enzymes were listed as main players, such as fatty acid synthase beta subunit dehydratse (*FAS1*), fatty acid synthase subunit alpha (*FAS2*), acetyl-CoA carboxylase [27] and 3-oxoacyl-(acyl-carrier-protein) synthase II (*FabF*). Hence, we proceeded to dissect the role of the *FAS1* gene in the lipid biosynthetic pathway of the multicellular *M. oryzae* and identify the processes it controls [14,28]. Lipid biosynthesis provides cells the ability to assimilate two-carbon compounds into the tricarboxylic acid (TCA) cycle and initiates fatty acid degradation to produce glycerol and fatty acids [29]. Fatty acid synthase subunit beta dehydratase catalyzes the formation of long-chain fatty acids from acetyl-CoA, malonyl-CoA and NADPH, which is the first step in the fatty acid biosynthesis [30]. 

In order to investigate the role of fatty acid biosynthesis in appressorium-mediated plant infection of *M. oryzae* S6, we first identified the copy number of *FAS1* locus (MG_04118) in the *M. oryzae* genome through analyses of the sequence deposited in Broads Institute (http://www.broadinstitute.org/). We identified only a single copy of this gene [5] in the database. The sequence for the *FAS1* gene was used to design primers for the amplification of the *FAS1* gene from our isolate. The amplified, purified and sequenced putative *FAS1* gene of *M. oryzae* S6 is approximately 6363 base pairs (bp) long (MT787294) and encodes a 2120 amino acids-long protein with a predicted molecular weight of 235.9 kDa (Supplementary Figure S1A,B). InterProScan identified the acyl transferase domain superfamily, acyl hydrolase, hydrolase, aldolase-type triosephosphate isomerase (TIM) barrel, hotdog domain superfamily, fatty acid synthase beta subunit, starter unit: acyl-carrier-protein (ACP) transacylase, fatty acid synthase, domain of unknown function DUF1729, *N*-terminal of enoyl-CoA hydratase (MaOC)-like dehydratase domain, acyl transferase, polyketide synthase and acyl transferase domain as the major homologous superfamilies and domains found in the FAS1 protein. 

**Figure 2:**
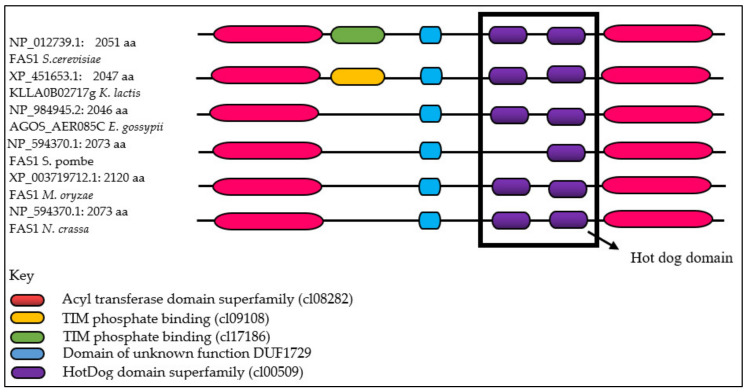
Proteins of Ascomycota identified as putative homologs of *Magnaporthe oryzae* strain S6 FAS1. The conserved domains and superfamilies of 5 homologous of AscomycotaFAS1 *Saccharomyces cerevisiae*, uncharacterized protein *Kluyveromyces lactis*, AER085Cp *Eremothecium gossypii*, FAS1 *Schizosaccharomyces pombe*, FAS1 *Neurospora crassa* and FAS1 *Magnaporthe oryzae* S6) were analyzed using InterProScan and pfam. Hotdog domain is boxed.

Multiple alignment analysis (Supplementary Figure S2) [31,32,33,34] of *M. oryzae* S6 FAS1 shows homology to FAS1 homologs and other related proteins of the fatty acid biosynthetic pathway of other filamentous Ascomycetes, such as *Saccharomyces cerevisiae* (FAS1, NP_012739.1, 56.7% identity), *Kluyveromyces lactis* (uncharacterized protein, XP_451653.1, 57.3% identity), *Eremothecium gossypii* (AER085Cp, NP_984945.2, 56.2% identity), *Schizosaccharomyces pombe* (fas1, NP_594370.1, 54.8% identity) and *Neurospora crassa* (FAS1, NCU07307, 78.4% identity) [35]. The comparison is based on conserved domains present between species. The conserved domains between the five homologous species determined from reverse psi-blast (rpsblast) search are: DUF1729 (pfam08354), TIM_phosphate_binding (cl09108), hot_dog (cl00509), acyl_transf_1 (cl08282) and TIM_phosphate_binding (cl17186) (Figure 2). Unknown function (DUF1729), TIM_phosphate_binding (cl09108), TIM_phosphate_binding (cl17186) and acyl_transf_1 (cl08282) domains have been identified in most fungi and bacteria but have not been linked to fatty acid metabolism. The HotDog domain, however, is involved in transcriptional regulation of fatty acid biosynthesis [36,37]. This domain is an ancient and ubiquitous motif, which is found in numerous prokaryotic, eukaryotic and archaeal organisms. This fold acts as an organic non-protein compound that binds with enzymes involved in cellular processes [38,39]. HotDog has a strong association with the regulation of lipid metabolism and cellular signaling, where proteins containing this fold are predominantly coenzyme A-binding enzymes [38,40]. Initially, the HotDog fold was identified in *Saccharomyces cerevisiae* [41]. The discovery of this domain in the FAS1 gene of our isolate indicates a possible role in fatty acid synthesis [39]. 

**Figure 3:**
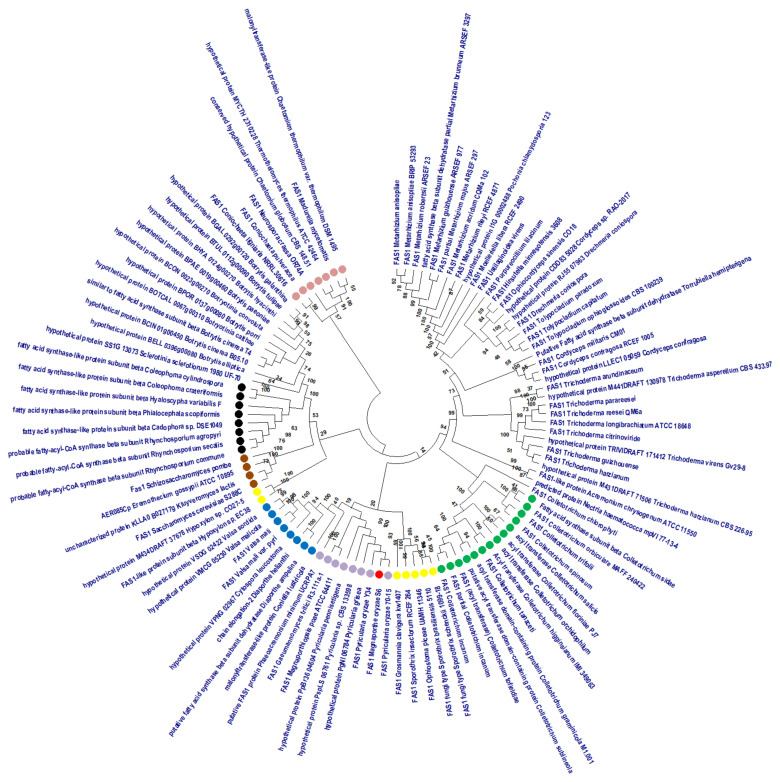
Molecular phylogenetic analysis by Maximum Likelihood method with bootstrap value on the branches. The evolutionary history was inferred by using the Maximum Likelihood method and Jones-Taylor-Thornton (JTT) matrix-based model [46]. The bootstrap consensus tree inferred from 1000 replicates is taken to represent the evolutionary history of the taxa analyzed [47]. Branches corresponding to partitions reproduced in less than 50% bootstrap replicates are collapsed. Initial tree(s) for the heuristic search were obtained automatically by applying Neighbor-Join and BioNJ algorithms to a matrix of pairwise distances estimated using a JTT model, and then selecting the topology with superior log likelihood value. This analysis involved 105 amino acid sequences. All positions with less than 95% site coverage were eliminated, i.e., fewer than 5% alignment gaps, missing data and ambiguous bases were allowed at any position (partial deletion option). There were a total of 2076 positions in the final dataset. Evolutionary analyses were conducted in Molecular Evolutionary Genetics Analysis Version 10 MEGA X [48].

A circular phylogenetic tree was assembled using 105 full sequences of FAS1 protein from the National Center for Biotechnology Information (NCBI) database (Figure 3). *M. oryzae* S6 (red) shares an ancestry with FAS1 from *Pyricularia grisea* Y34 (*P. grisea*), and *Pyricularia oryzae* strain 70-15 (*P. oryzae*), hypothetical *protein Pyricularia pennisetigena* (*P. pennisetigena*)*,* hypothetical protein *Pyricularia sp*., *Gaemannomyces tritici* (*G. tritici*) and *Magnaporthiopsis poae* (*M. poae*) (purple). These species with shared ancestry are plant pathogenic filamentous fungi that cause widespread disease and losses in cereals, grass crops, wheat and rice. *M. poae* and *G. tritici* produce sexual structures, perithecia, asci and ascospores, but *M.oryzae* and Pyricularia species produce bear spores sympodially on conidiophores [31,42,43]. *Pycularia species* and *M. oryzae* target the leaf, node, neck, grain and collar of rice plants, unlike *M*. poae** and *G. tritici,* which are soil-borne necrotrophic parasites that infect roots of grasses resulting in root rot and subsequently, host-plant death [31,33]. Magnaportheceae, such as *M. oryzae, G. titici, M. poae* and *P. oryzae,* which initially infected rice, have evolved and started infecting grasses, wheat and barley [43,44,45]. This Magnaportheceae family species has travelled from Eastern Asia to Southeast Asia, then to South America and North America, as seen in Supplementary Figure S3.

Fungus under genus *Coniella, Valsa* and *Diaporthe* are closely related to FAS1 *M. oryzae* (blue), and are found in Western Europe, Eastern Asia and Northern America (Supplementary Figure S3). Magnaportheceae share ascospore morphology with Schizoparmeaceae, Valsaceae and Diaporthaceae families [49]. FAS1 of *Phaeacremonium minimum* (pink) is closely related to FAS1 *M. oryzae* S6, where Phaeoacremonium and Magnaportheceae share disease symptoms including vascular necrosis, color change on leaves, wilting and subsequent death [50]. Another branch of the same clade consists of *Coniochaeta, Chaetomium, Thermothelomyces Madurella* and *Neurospora* (pink), which share the chitin synthase pathway that plays a role in the virulence of these fungal phytopathogens. *Coniochaeta* and *Chaetomium* shares the same characteristics with *Magnaporthe* genus in terms of genetic capability to produce at least two types of melanin and to facilitate protein expression on different types of carbon sources [51]. Meanwhile, *Thermothelomyces* and *Neurospora* genus only exhibit similarities as ascomycetes and in conidia structures with *M. oryzae* [52,53]. The five families in this branch are mainly found in North America and Africa. 

The neighboring clade to FAS1 *M. oryzae* S6 has the *Colletotrichum* sp. (green) [54,55], which differentiates specialized appressoria to breach the plant’s cuticle [55]. This plant pathogen shares common G1 phase that controls and coordinates appressorium development [55]. The other neighboring two clades to *M. oryzae* S6 FAS1 consist of four different genus’, which are *Sporothrix, Ophiostoma, Hypoxylon* and *Grosmannia* (yellow), that have no reports of similarities with *Magnaporthe* except in protein sequence [56]. Many intrinsic characteristics of these fungal pathogens allows for survival within the host through adherence to host cells, the secretion of extracellular hydrolytic enzymes, morphogenetic switching and fungal stress responses [57]. Geographical distributions of these monophyletic groups are focused in Japan (Southeastern Asia), Europe and America. Lastly, another five different genus, *Coleophoma, Haloscypha, Phialocephala, Cadophora* and *Rhynchosporium* (black), are in a separate clade and are mainly focused in Southeast Asia. This group is known as root-associated fungi and is linked to various lifestyles, including saprobes, bryophilous fungi, root endophytes, as well as symbionts [58]. The *FAS1* gene from four different families, highlighted in brown, represent putative homologs of *M oryzae* strain S6 FAS1. The conserved HotDog domain was found in these four homologs. In conclusion, the organisms in neighboring clades of FAS1 *M. oryzae* S6 though originating from different geographical locations and different genus have similarities in the infection processes of the host and in terms of structures produced for infection. The Ascomycetes discussed above were isolated from host such as plants, animals, insects and even humans. Majority of the host are plants, such as cereals, millet, flowering plants, fruit plants, decaying wood and grass. Highlighted features among the hosts are high content of carbon source, lipids, fatty acids, glycerol, glycogen and others [31,42,43,58]. Summary of ascomycetes fungus strain, phylum, substrate, host and country which are closely related to *Magnaporthe oryzae* S6 Malaysian strain shown in Supplementary Table S2.

### 2.2. Generation of FAS1 Mutants

**Figure 4:**
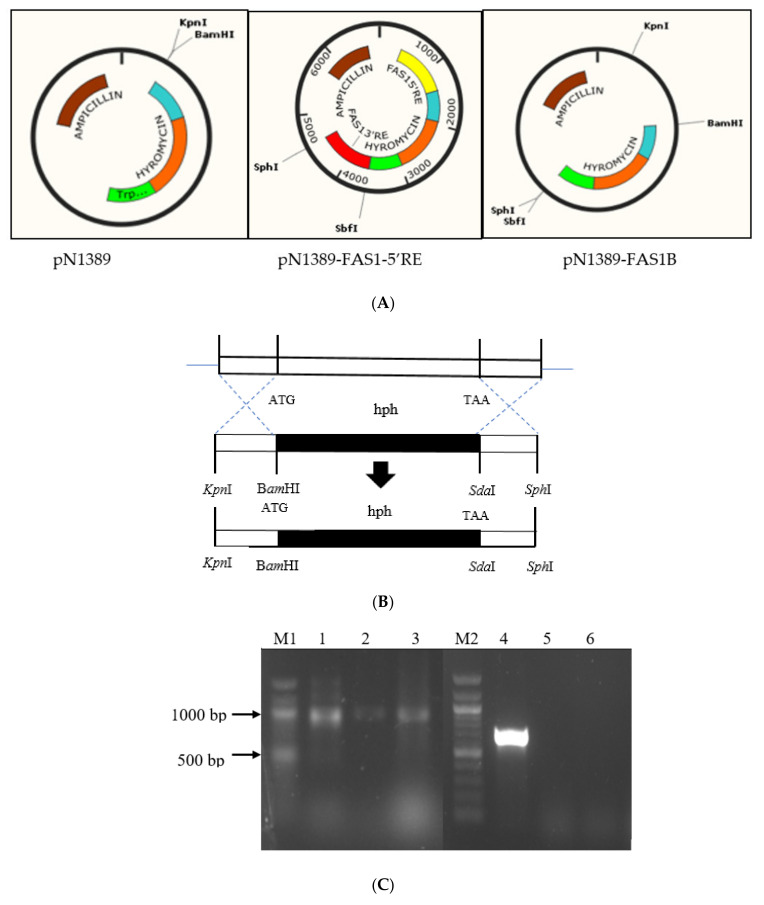
Schematic presentation of the FAS1 gene deletion process through homologous double crossover to generate the pN1389-FAS1 construct. Validation of putative transformants and protein visualization is provided. (**A**) Schematic diagram outlining the ligation of 5′ FAS1 and 3′ FAS1 with restriction sites into pN1389 plasmids. (**B**) Assembly of pN1389-FAS1-5′RE and pN1389-FAS1 formation. (**C**) Validation of mutants with hygromycin and fas1 gene primers. Lane M1 and M2: 1 kb ladder (Biolab, England), (a) Lane 1: pN1389 plasmid as a positive control (~1000 bp), (b) Lane 2: Transformant fas1-1 amplified with hygromycin primers (~1000 bp), (c) Lane 3: Transformant fas1-2 amplified with hygromycin primers (~1000 bp), (d) Lane 4: Genomic DNA wildtype amplified with fas1 gene primers (700 bp), (e) Lane 5: Transformant fas1-1 amplified with fas1 gene primers (no results), (f) Lane 6: Transformant fas1-2 amplified with fas1 gene primers (no results). Protein visualization via SDS-PAGE provided in the Supplementary Figure S4.

To knockout the *FAS1* gene in *M. oryzae* S6, its upstream and downstream flanking sequence were amplified with primer pairs FAS1pN5 Forward/FAS1pN5 Reverse and FAS1pN3 Forward/FAS1pN3 Reverse with restriction sites *Kpn*I, *Bam*HI, *Sda*I and *Sph*I inserted, respectively (Supplementary Table S1). The construction of expression vectors by the homologous double crossover method was achieved by assembling within the hygromycin cassette containing pN1389 vector, the 5′ and 3′ flanking region of *fas1* sequence of *M. oryzae* S6 before the glucoamylase promoter and TrpC terminator were included [59,60]. Insertion of the 5′ flanking region (~1000 bp) into its multiple cloning site between the promoter and ampicillin resulted in pN1389-FAS1-5′RE. This was then followed by an insertion of the 3′ flanking region into the multiple cloning site between terminator and ampicillin to generate pN1389-FAS1 cassette (Figure 4A). The schematic diagram of the overall strategy to generate the knockout vector of *FAS1* gene is as shown in Figure 4A,B. Through this method, a 5.8 kb portion of the 6.3 kb coding sequence of *FAS1* was replaced with a 2 kb hygromycin resistance cassette (Figure 4B). The resulting cassette was introduced into *M. oryzae* S6 spheroplasts. Two hygromycin-resistant transformants (*Δfas1-1* and *Δfas1-2*) were isolated and screened by polymerase chain reaction (PCR) verification (Figure 4C) using primers designed to amplify the hygromycin gene (~900 bp) (hygromycin (HYH) Forward/ HYH Reverse) and *fas1* primers (~700 bp) (FAS1A Forward/FAS1A Reverse). Protein extracted from the transformed *M. oryzae* S6 and wildtype S6 were fractioned through Sodium Dodecyl Sulfate–Polyacrylamide Gel Electrophoresis (SDS-PAGE). The predicted approximate 235 kDA protein was not observed in the mutants (*fas1-1* and *fas1-2*), indicating a successful deletion of the *FAS1* gene in the transformants (Supplementary Figure S4) [61].

### 2.3. Carbon Source Utilization Altered in fas1 Mutants.

Unsaturated fatty acids are categorized into short, medium, long and very long-chain fatty acids [62,63]. Metabolism of short chain fatty acids (1–6C) and medium chain fatty acids (7–19C ) occurs through the process of β-oxidation, which takes place in the peroxisomes before lipid biosynthesis [64,65]. Peroxisomal metabolic function was evaluated utilizing either short chain fatty acids (acetic acid and glucose) or medium chain fatty acids (oleic acid and olive oil) in Δ*fas1-1 and Δfas1-2* mutants. Vegetative growth rate between mutant and wildtype *M. oryzae* S6 on various media was examined and the diameter was measurements in 10-day-old cultures of wildtype, Δ*fas1-1* and Δ*fas1-2* mutants in three biological replicates (Complete Media (CM), Potato Dextrose Agar (PDA), Minimum Media (MM) + glucose, MM + olive oil and MM + acetic acid) at 26 °C (Supplementary Table S3). 

**Figure 5:**
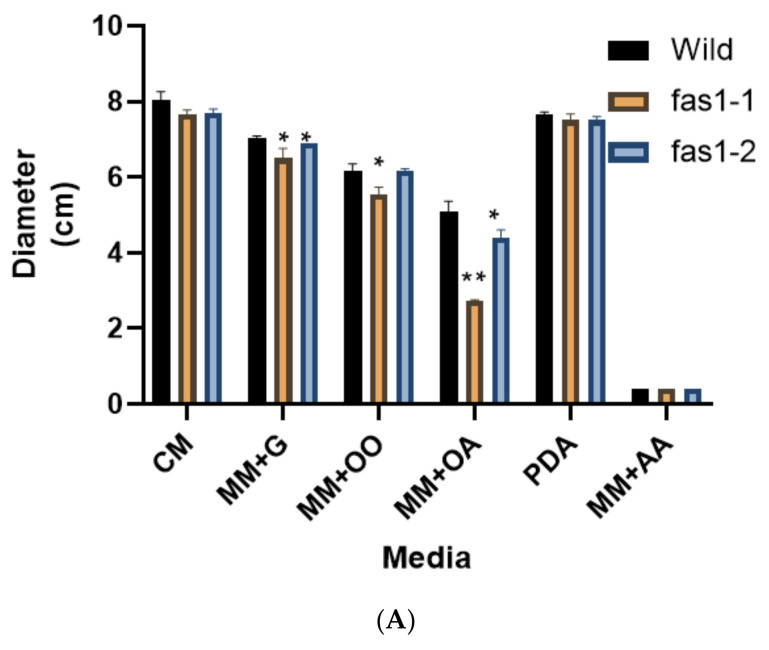

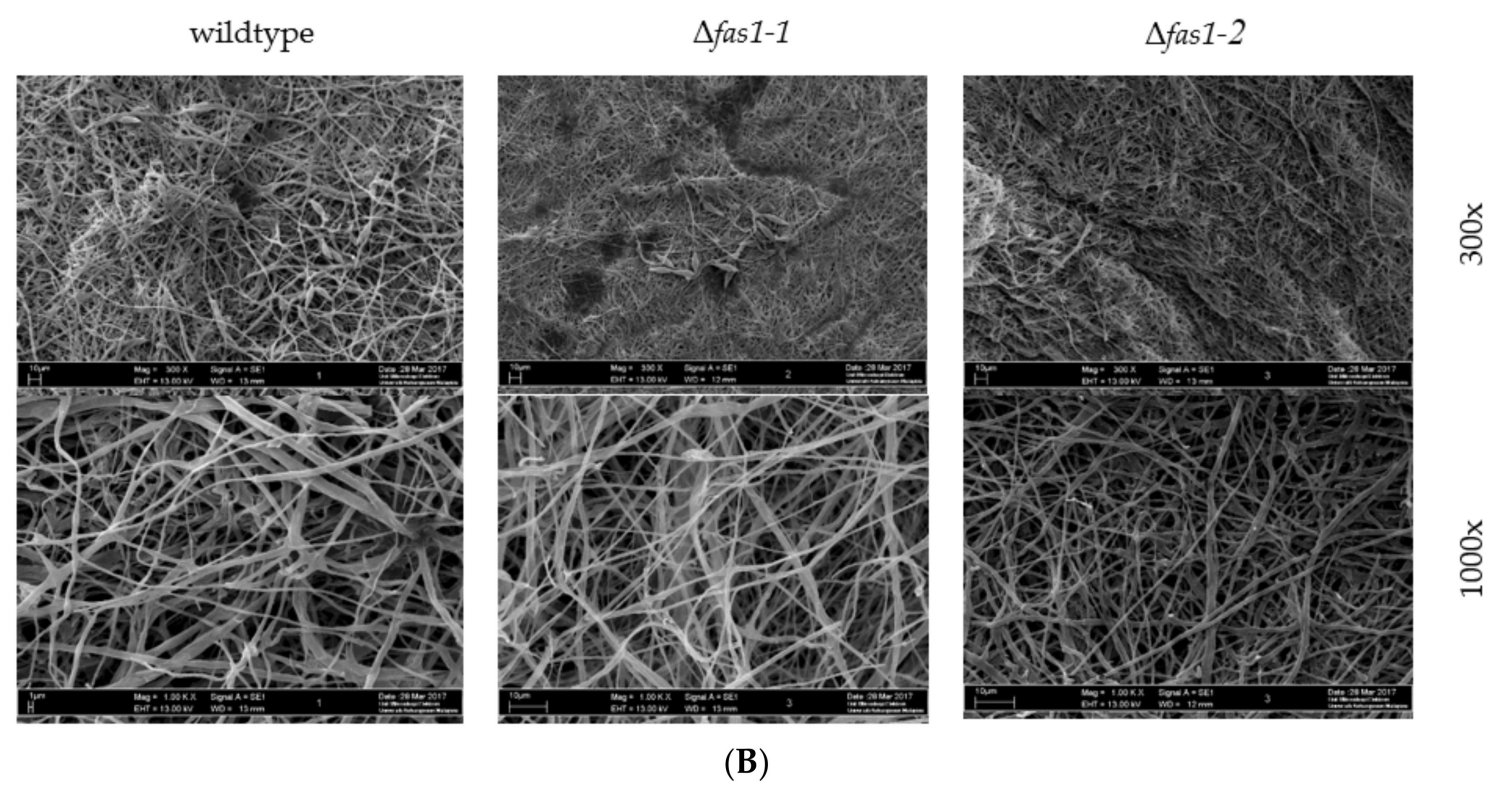
Growth rate and mycelial structure under scanning electron microscope (SEM). (**A**) Growth rate on different carbon source of wildtype, *fas1-1* and *fas1-*2 after 10 days of incubation on complete medium agar plates at 26 °C. Error bars represent the standard deviation. Single asterisk represents significant difference (*p* ≤ 0.05). Double asterisks represent significant difference (*p* ≤ 0.01). (**B**) image of wildtype, *fas1-1* and *fas1-2* mycelium structure after 10 days of incubation on complete medium at temperature of 26 °C. Magnification 300× and 1000× taken using SEM.

Statistical analyses showed significant differences (*p* < 0.05) in the growth rate between wildtype, Δ*fas1-1* and Δ*fas1-2* mutants. The observed growth rate was lower in Δ*fas1-1* mutants on CM, MM + glucose and MM + olive oil, with 5 to 10% reduction observed compared to wildtype. Meanwhile, Δ*fas1-2* mutants’ vegetative growth was reduced by 2 to 4% on CM, MM + glucose and MM + olive oil (Figure 5A and Supplementary Table S3). The colony diameter on MM + oleic acid showed the highest reduction (46% and 14%) in Δ*fas1-1* and Δ*fas1-2* mutants. Wildtype measured growth of 5.10 ± 0.26 cm compared to Δ*fas1-1* and Δ*fas1-2* mutants, with an average diameter of 2.73 ± 0.03 cm and 4.40 ± 0.20 cm, respectively (*p* < 0.05). Both mutants showed a reduction of 3% compared to wildtype on PDA. Cultures incubated on MM supplemented with 50 mM acetic acid showed no growth for wildtype, Δ*fas1-1* and Δ*fas1-2* mutants. 

**Figure 6:**
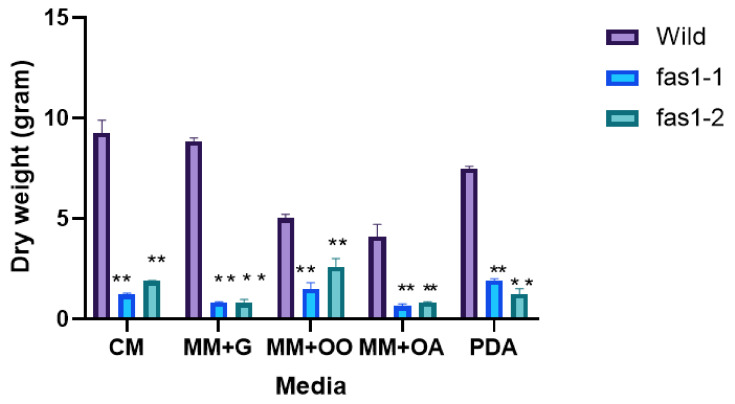
Dry weight assay of wildtype strain S6 compared to mutant. Dry weight of wildtype mycelium, *fas1-1* and *fas1-2* on five different carbon sources was taken 10 days post-growth at 26 °C. Error bars represent standard deviation from three biological replicates of this experiment. Double asterisks represent significant difference (** *p* ≤ 0.01).

**Figure 7:**
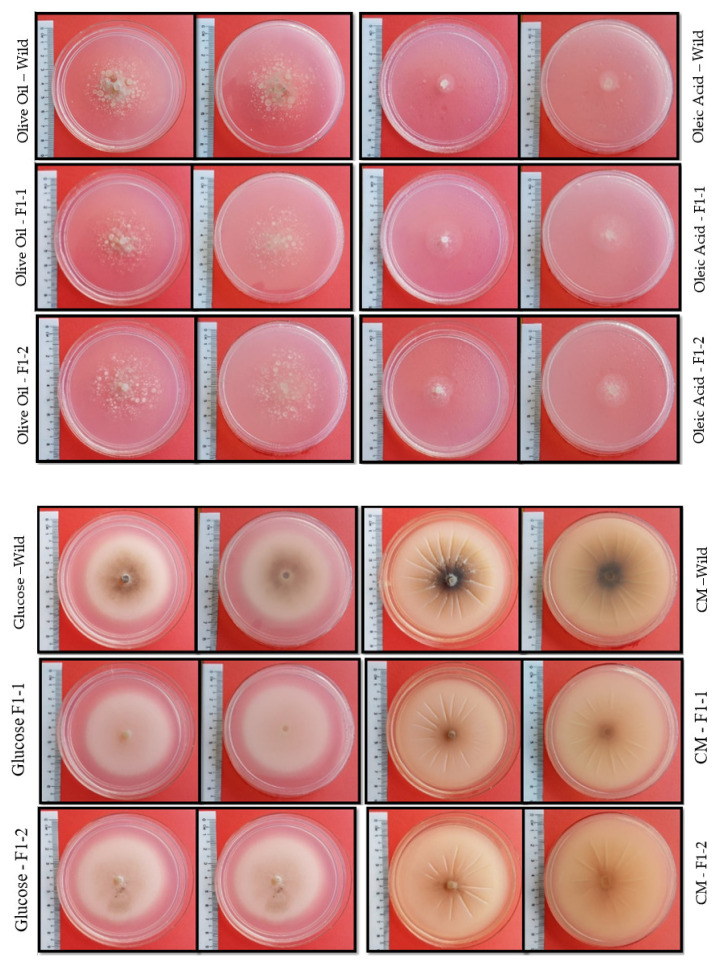

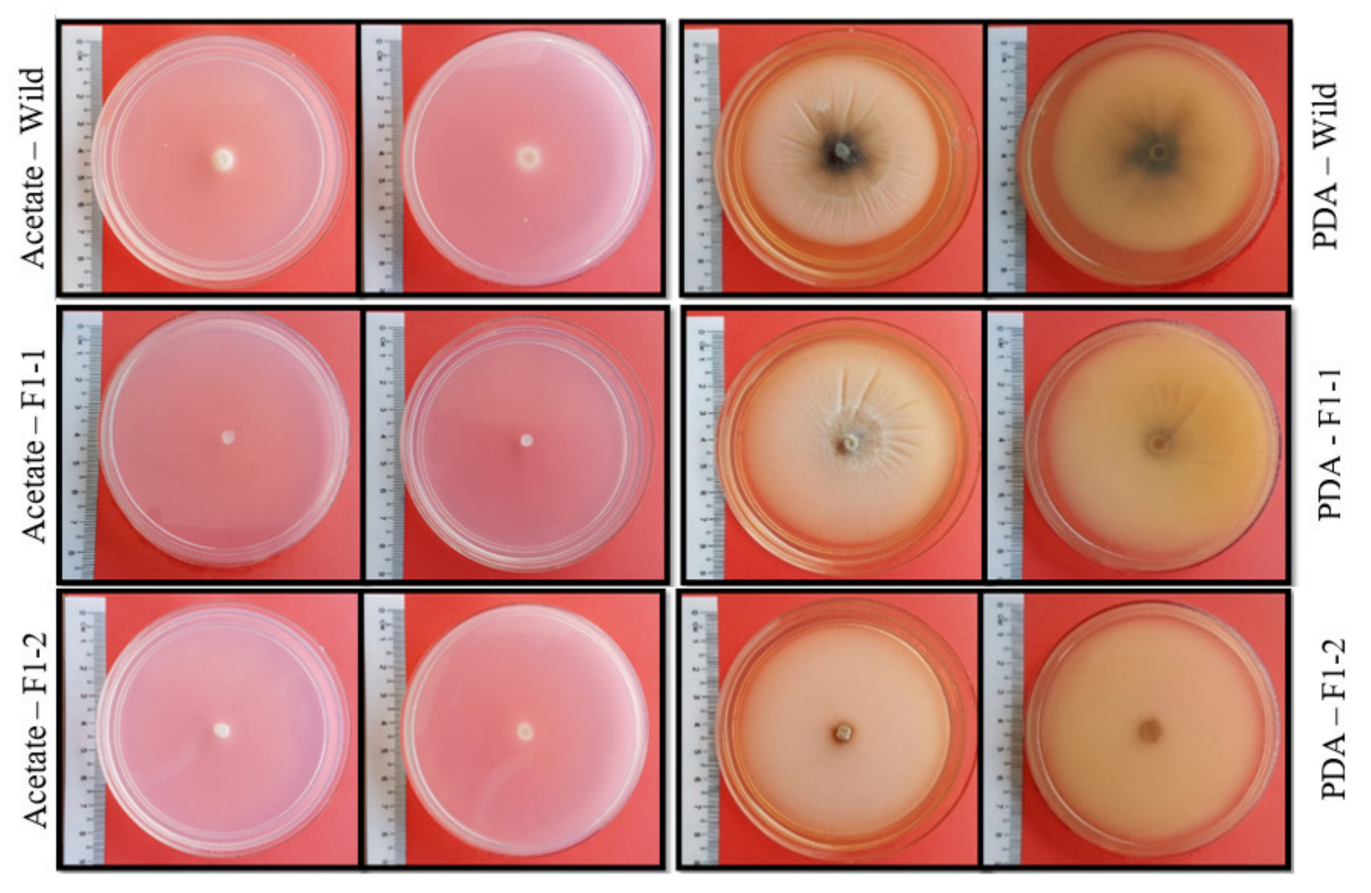
Pigments produced by wildtype and mutant *M.oryzae* S6 on different carbon sources. Appearance and vegetative growth of wildtype, *fas1-1* and *fas1-*2 after 10 days of incubation on different carbon sources at 26 °C. Δ*fas1-1* and Δ*fas1-2* mutants showed lighter colony pigmentation and light-brown, whitish pigments compared to the blackish-brown colonies of the wildtype *M. oryzae* strain S6 on CM, PDA, MM supplemented with 50 mM glucose and MM supplemented with 50 mM olive oil.

Further, scanning electron microscope (SEM) electrographs obtained on different media showed aerial hypha of Δ*fas1-1* and Δ*fas1-2* mutants as sparse and with thinner mycelium compared to the dense, compact aerial hypha and mycelium observed in wildtype (Figure 5B). In order to further explore the differences in vegetative growth of the Δ*fas1-1* and Δ*fas1-2* mutants, we also carried out mycelial dry weight assays on different carbon sources. The results showed that Δ*fas1-1* and Δ*fas1-2* mutants’ mycelial dry weight on all carbon sources were significantly reduced compared to wildtype (*p* < 0.05). The results showed the highest drop on MM supplemented with 50 mM glucose in *Δfas1-1* mutants at 91% and 92% (Δ*fas1-2*) (Supplementary Table S4 and Figure 6). The least reduction was observed on MM supplemented with 50 mM olive oil, where Δ*fas1* mutants showed reduced growth by up to 70% and 48%, respectively (Supplementary Table S4 and Figure 6). The difference in growth diameter measured is significantly different between wildtype and mutants, where oleic acid showed the most reduction in Δ*fas1-1* mutants. Acetic acid is not a preferred fatty acid source for *M. oryzae* according to the growth profile (Figure 5, Figure 6 and Figure 7). High-concentration acetic acid seemed to affect fungal growth of *M. oryzae* S6. In Figure 5, growth of wildtype and mutants were hampered by addition of acetic acid into media. This is clearly seen by the absence of any radial mycelial growth surrounding the mycelial plugs of *M.oryzae* in Figure 7. In a previous study, acidification due to low concentration of acetic acid (≤1 µM) was shown to occur at the tip of germ tubes that differentiate, grow and develop by mitosis to create somatic hyphae that induce appressorium formation in *M. oryzae* Δ*icl* mutants. This evidence suggests that a low concentration of acetic acid activates glycoxylate cycle and promotes faster appressorium differentiation. However in this study, the high concentration of acetic acid (≥1 µM) that alters pH significantly, causing suppression of growth of germ tube and mycelium [66]. 

In *Magnaporthe*, the degradation of lipid reserves remains an important role in turgor pressure generation in appressorium during infection [67]. Across different organisms, including yeast, bacteria and fungi, fatty acid β-oxidation occurs primarily within the peroxisomes. However, very little is known of the relationship between the peroxisomal fatty acid metabolism and the lipid biosynthetic pathway. Studies show fatty acid catabolism occurs at peroxisomes and mitochondria during infection process through the β-oxidation pathway that initiates lipid biosynthesis in most pathogenic fungi, such as *M. oryzae*, *Heterobasidion annosum, Candida glabrata* and *Cryptococcus gattii* [68,69]. Metabolic function of peroxisome plays an important role in fungal development and pathogenesis. Fatty acid β-oxidation metabolism produces acetyl-CoA that acts as a precursor in the glyoxlate, gluconeogenesis and lipid biosynthesis cycles involved in producing necessary cellular metabolites [64,68,70,71]. Multi-functional proteins (MFPs), which are involved in the second and third step of fatty acid β-oxidation cycle, results in *Δmfp1 mutants* that are unable to grow on olive oil and oleic acid, consistent with an inability to metabolize fatty acids. MFP1 is highly expressed in spores and vegetative mycelium in the presence of olive oil and oleic acid. The vegetative growth of our mutants on olive oil and oleic acid was reduced compared to wildtype, suggesting that the deletion of FAS1 may have slightly impaired the fatty acid β-oxidation metabolism in *M. oryzae* S6 necessary for lipid utilization [14]. 

A significant reduction in dry mycelial weight was observed between wildtype compared to mutant strains. This could be attributed to the visualized, denser more compact SEM micrograph of wildtype compared to mutants. The reduced dry mycelial weight, and less dense and compacted SEM visualization of mycelium in mutants, indicate that the mycelial cell wall composition and integrity may have been affected. Fungal cell walls are composed of polysaccharides and mannoproteins that make up the chitin and glucan components of these walls [72]. Chitin is an integral part of fungal cell wall and depends on the activity of chitin synthase enzymes [73]. MoGls2, a yeast glucosidase II homolog in *M. oryzae*, is required for trimming of the final glucose in N-linked glycans, and in normal cell wall synthesis. *ΔMogls2* mutants have defective cell walls and cell wall integrity, resulting in lower protoplast production in these mutants. This indicates that MoGls2 plays an important role in cell wall integrity in *M. oryzae* [74]. The reduction in dry mycelial weight and the fine mycelium structure of *Δfas1-1* and Δ*fas1-2* is a consequence of changes to the cell wall composition in the mycelium, which stipulates a possible role for *FAS1* in cell wall synthesis of *M. oryzae* (Figure 5B). 

### 2.4. Mutants Display Reduced Pigmentation

**Figure 8:**
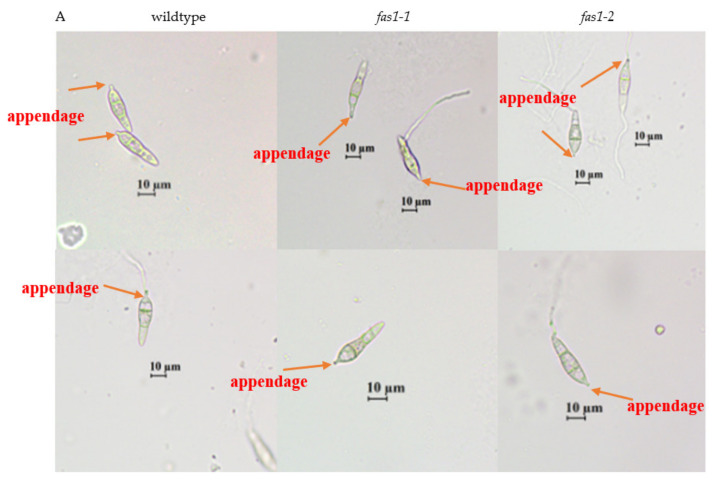

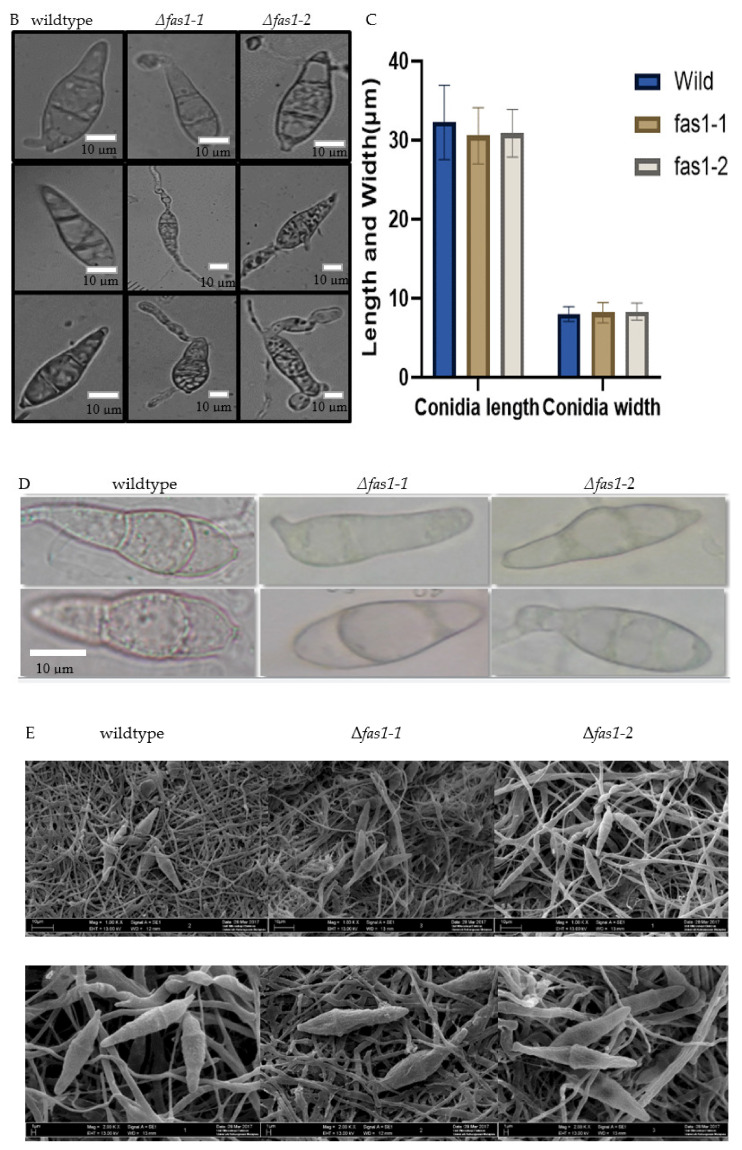
Effect of *FAS1* deletions on conidiogenesis, appressorial formation and vegetative growth. (**A**) Light microscopy of conidia cultured on oatmeal agar 10 days post-incubation at 26 °C. Basal appendage formation observed where conidia is attached to conidiophores. Magnification 100× light microscope. Bar = 10 µm. (**B**) Appressorium formation of conidia inoculated onto plastic cover slips in a moist chamber at 24 °C. (**C**) Size measurement of conidia length and width in micrometer, 10 days post-inoculation at temperature of 26 °C. Error bars represent the standard deviation. (**D**) Appearance of wildtype, mutant *fas1-1* and mutant *fas1-2* spore formation after 10 days of incubation on complete medium at temperature of 26 °C. Magnification 100x light microscope. (**E**) Scanning electron microscope image of wildtype, mutant *fas1-1* and mutant *fas1-2* spore after 10 days of incubation on complete medium at temperature of 26 °C. Magnification 1000× and 2000× of scanning electron microscope.

Deletion of the *FAS1* gene showed some changes in the colony pigmentation in *Δfas1-1* and *Δfas1-2* mutants of *M. oryzae* strain S6 (Figure 8). Comparisons were made between 10-day-old cultures. Compared to the wildtype strain of *M. oryzae* S6, *Δfas1-1* and *Δfas1-2* mutants formed mycelium that was not well pigmented (Figure 7). CM, PDA, MM supplemented with 50 mM glucose and MM supplemented with 50 mM olive oil media were used to grow the Δ*fas1-1* and Δ*fas1-2* mutants. The mycelia of Δ*fas1-1* and Δ*fas1-2* showed lighter colony pigmentation compared to the blackish-brown colonies of the wildtype. More and deeper radial lines were formed on the surface of colonies of PDA and CM media of wildtype *M. oryzae* strain S6 compared to Δ*fas1-1* and Δ*fas1-2* mutants’ colonies (Figure 7). This observation indicates that the mutants may be experiencing a reduction in melanin production. This is the first report on the effect of *FAS1* gene deletion on *M. oryzae* S6 and its possible role in melanin pigment development. 

The appressorium has a differentiated strong cell wall, enriched in chitin and layer of melanin formed between the cell wall and cell membrane and acts as a barrier to the efflux of glycerol during turgor pressure generation [75,76]. Accumulation of glycerol generates hydrostatic turgor pressure. Lipid bodies are mobilized during appressorium formation and are simultaneously oxidized by β-oxidation into acetyl-CoA. Acetyl-CoA is used in the secondary metabolite pathways, such as melanin and polyketide biosynthesis. Melanin, polyketide and lipid biosynthesis triggers turgor generation [76]. In a little over a decade, researchers have studied genes that have been linked to lipid biosynthesis, melanization and pathogenicity of fungi. Two such genes linked to these processes are the peroxisome biogenesis factor (*PEX*) and the isocitrate lyase (*ICL*). 

**Figure 9:**
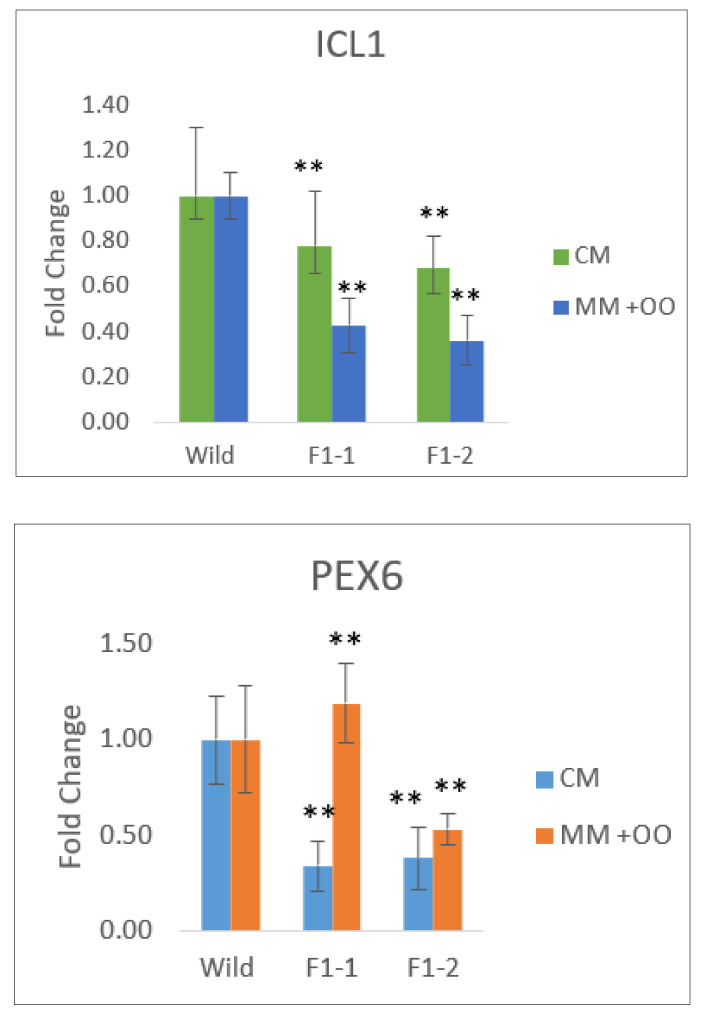
Gene expression of *PEX6* and *ICL1* expression in the wildtype strain S6, *Δfas1-1* and *Δfas1-2* mutants grown in complete media for 48 h and then transferred to a new flask of complete medium, or minimal medium containing 50 mM olive oil for 24 h. Expression profiles of *PEX6* (peroxisomal biogenesis) and *ICL1* (glyoxylate cycle) genes were analyzed. Gene expression data of *PEX6* and *ICL1*, obtained from quantitative RT-PCR analysis, were normalized by using with housekeeping gene *EF1α* as an internal control, calculated using three independent biological experiments with three technical replicates for each independent experiment, as reported above. Double asterisks represent significant difference (** *p* ≤ 0.01).

The *PEX* gene is associated with lipid degradation, appressorial melanization and turgor genesis. Studies conducted on Δ*mopex13* and Δ*mopex14* mutants in *M. oryzae* demonstrated reduced growth, lighter colored appressoria, defective melanization, appressorial glycerol accumulation and finally, its inability to infect host in pathogenicity test. Δ*mopex13* and Δ*mopex14* mutants were also unable to degrade fatty acids in their spore [75]. PEX13 in *Colletrichum orbiculare* and *Aspergillus nidulans* showed defective melanization and loss of ability to penetrate the cuticle of the host cells [19,75]. Disruption of PEX7 blocked the PTS2 import pathway in *M. oryzae* that is presumed to connect lipid biosynthesis, melanin biosynthesis and interruption of lipid degradation [75]. Δpex6 mutants showed that this gene was crucial in lipid body degradation and melanization. From the previous reports on the *PEX* gene, it may be concluded that it is part of the machinery involved in degradation of fatty acid and melanization. Therefore, from what is seen in Figure 7, Figure 8 and Figure 9, we can conclude that the *FAS1* mutants may have altered function in melanin biosynthetic pathway, as observed in the light pigmented Δ*fas1-1* and Δ*fas1-2* mutants. 

Lipid biosynthesis (lipogenesis) takes place in the cytoplasm where acetyl-CoA is used as the precursor [77]. The *FAS1* gene is involved in converting acetyl-CoA into malonyl-CoA [28]. Deletion of ICL leads to a reduction in pathogenicity due to the connection between lipid biosynthesis pathway and glyoxylate through acetyl-CoA [29]. Fungal pigmentation is regulated through polyketide biosynthesis. Polyketides biosynthesis involves repetitive condensation reactions that involves acyl groups derived from acetyl-CoA [78]. Again, acetyl-CoA connects the melanin pathway (polyketide pathway), lipid biosynthesis and the glyoxylate pathway. This relationship predicts the connection between pathogenicity and melanin biosynthesis of fungi through the *ICL* gene. In a previous study, the Δ*ICL1* mutant had significantly delayed germination and reduced pigmentation. It is possible that the reduction in *ICL1* expression in Δ*fas1-1* and Δ*fas1-2* is attributed to interference in the above pathways that leads to disruption in melanin production, lipid biosynthesis and pathogenicity [79]. *FAS1* gene deletion experienced the same results with *ICL1* mutants, where it showed reduced pigmentation on different carbon sources (Figure 8 and Figure 9). 

Carbon and nitrogen enhance the production of polyketides that are involved in the melanin biosynthetic pathway. In previous studies, glycine (nitrogen source) showed the highest value to enhance production of secondary metabolites. PDA and CM media contain glycine as a nitrogen source and in combination with carbon sources such as glucose (CM) and dextrose (PDA), darker melanin formation should be observed compared to MM supplemented with 50 mM glucose and 50 mM olive oil [80,81].

### 2.5. Functional FAS1 Gene Needed for Conidiogenesis

To investigate the role of fatty acid mobilization during appressorium formation, we first studied the ability of wildtype strain S6, *Δfas1-1 and Δfas1-2* mutants to form spores by collecting them from the surface of 10-day-old cultures on oatmeal and PDA plates using sterile distilled water. Oatmeal and PDA were used as these media induced spore production in *P. oryzae* and *M. oryzae* [82,83]. Δ*fas1-1* and Δ*fas1-2* mutants exhibited reduced conidial formation (oatmeal: *fas1-1* 0.25 ± 1.70 × 10^4^ cell/mL, *fas1-2* 0.5 ± 2.4 × 10^4^ cell/mL; PDA: *fas1-1* 1.0 ± 1.50 × 10^4^ cell/mL, *fas1-2* 1.0 ± 2.11 × 10^4^ cell/mL), whereas the wildtype strain S6 produced numerous conidia with 2.0 ± 0.5 × 10^4^ cell/mL and 5.0 ± 2.1 × 10^4^ cell/mL respectively, on oatmeal and PDA (Supplementary Table S5). Results from these conidiation assessments showed that the deletion of *FAS1* triggered a ~10-folds reduction of conidiation on oatmeal media and a ~5-folds reduction on PDA media (Supplementary Table S5). 

A detailed microscopic observation of the infection process was undertaken to determine which stage of conidiogenesis was affected in the Δ*fas1* mutants. The Δ*fas1* mutants produced less conidia and it was abnormally shaped. From microscopic observations, the basal appendage formation in wildtype *M. oryzae* S6 and Δ*fas1* mutants were similar (Figure 8A). The wildtype and Δ*fas1* mutants are able to attach to conidiophores as the basal appendage formation is not disrupted by *FAS1* deletion. Although the septum and basal appendage formation of mutants is normal under light microscopy, the constriction site at the base of the mutant spore is incomplete, and therefore may result in loose grip of conidia to the conidiophore (Figure 8A). Obvious morphogenetic defects were observed in the sporulation of the mutants, causing a complete block in the formation of conidiophores, which is essential for the pathogenicity of fungi [84]. The lengths of conidia of Δ*fas1-1* and Δ*fas1-2* mutants were 30.6 ± 3.6 µm and 31.0 ± 3.1 µm respectively, which was slightly shorter than that of wildtype at 32.2 ± 4.7 µm (Supplementary Table S6 and Figure 8C). The width of conidia of wildtype at 8.1 ± 0.9 µm and Δ*fas1* mutants at 8.2 ± 1.3 µm and 8.3 ± 1.1 µm, demonstrated no significant difference (*p* > 0.05) (Figure 8C). Supplementary Tables S6 and S7 provides the lengths and widths of the conidia of 10-day-old cultures grown under a 12 h day/12 h dark period on complete media and calculation of significant value. The appressorium formation of conidia in *Δfas1-1* and *Δfas1-2* mutants was abnormal; specifically, in septa formation, the distribution of the area between the septa and overall lipid distribution is altered compared to wildtype (Figure 8B,D). Impaired lipid distribution and septa formation resulted in abnormal shaped spores (Figure 8). Mutants produced elongated shaped conidia, compared to the pyriform-shaped wildtype conidia.

In *M. oryzae*, MoAND1 mediates positioning of fungal nuclei during asexual reproduction and the initial penetration phase of fungal pathogenesis. In a previous study, deletion of MoAND1 in *M. oryzae* displayed abnormalities in septation pattern and lipid distribution, resulting in the inability to germinate. Δ*Moand1*, decreased ∼65% of conidia formation compared to wildtype [85,86]. SEM visualization (Figure 8E) revealed abnormal spore formation with less septae, and asymmetrical teardrop-shaped spores. Δ*Moand1* and Δ*fas1* mutants exhibit high similarity as both have impaired lipid distribution and septae formation in spores (Figure 8B) [86]. As seen in the mutant spores, lipid deposits are largely reduced or not present. This indicates that the abnormal spore and appressorium formation observed in the Δ*fas1-1* and Δ*fas1-2* mutants may be a consequence of a defective lipid biosynthetic pathway.

According to Figure 1, if the lipid biosynthetic pathway is blocked by deletion of *FAS1,* there are three other pathways that can produce lipid for appressorium formation, but the amount of lipid produced is less. Transport of lipid reserves have been reported in the maize pathogen *Colletotrichum graminicola* via a mechanical infection mechanism similar to *M. oryzae,* where they generate invasive forces in appressoria for penetration into the host [85]. Hence, the reduced observed lipid deposits can cause spore and appressorium formation to be impaired. From these results, we can presume that *FAS1* essentially regulates conidiophores development and conidiogenesis of *M. oryzae* S6.

The spores of *Δfas1-1* and *Δfas1-2* mutants exhibited distinct defects in conidiation, which is similar to that observed in *MoPer1*. MoPer1 is a homolog of *Saccharomyces cerevisiae* ScPer1, from rice blast fungus *M. oryzae*. The conidia produced by the Δ*Moper1* mutant were abnormal in morphology but formed normal germ tubes. Microscopic examination revealed that the rate of appressorium formation in *ΔMoper1* was significantly more reduced than wildtype strain, which was only about 10%, while the wildtype was more than 90%. Previous studies suggest that Δ*Moper1* plays a crucial role in conidiogenesis and appressorium formation. Based on our observation in this study, we can assume that *FAS1* may also be involved in the organization of sporulation and conidium morphology based on the structural defects observed in the mutants [87]. 

### 2.6. FAS1 Enzyme Activity Is Essential for Appressorium Formation

Deletion of *FAS1* interrupted FAS1 and FAS2 enzyme activity according to the result obtained following the enzyme assay. The enzyme-linked immunosorbent assay was used to detect enzyme activity of fatty acid synthase encoded by FAS1 and FAS2. Fatty acid synthase (FAS) enzyme activity was not detectable in Δ*fas1-1* and Δ*fas1-2* mutants compared to wildtype strain grown on CM medium (with glucose), which showed 0.363 ng/mL protein reading. Meanwhile, in MM supplemented with 50 mM olive oil medium, Δ*fas1-1* and *Δfas1-2* mutants and wildtype presented no activity (Table 1). 

FAR1 and FAR2 proteins of *M. oryzae*, encode highly conserved members of the Zn2-Cys6 family of transcriptional regulators. The *A. nidulans,* Δ*far1* and Δ*far2* mutants were unable to grow on medium fatty acids (7–19C ), and the same was observed in *M. oryzae,* where both *FAR1* and *FAR2* were independently required for the utilization of 7–19 C fatty acids [16,88]. However, Δ*far2* was unable to grow on short chain fatty acids, while Δ*far1* was able to utilize short chain fatty acids, hence *FAR2* is important for growth on both fatty acids. In this research, *FAS1* enzyme activity was not detected on media supplemented with glucose (1–6 C fatty acid) and olive oil (7–19 C fatty acid). These results suggest that the Δ*fas1* mutants may lack functional peroxisomes and may be defective in β-oxidation of fatty acids.

### 2.7. FAS1 Mutant Exhibited Reduction in Machinery Connected to Fungal Pathogenicity

We assayed the transcript abundance of *M. oryzae* genes associated with the melanin and lipid production pathway [16]. *PEX*6 and *ICL1* genes were selected for expression analysis in wildtype *M. oryzae* and *Δfas1* through real-time PCR to determine if there was any difference in the expression of these fatty acid utilization genes. Total RNA was isolated from wildtype strain S6, Δ*fas1-1* and *Δfas1-2* mutants grown on two media, CM medium with glucose and MM supplemented with 50 mM olive oil. The expression profiles of *PEX6* and *ICL1* genes were then determined, as shown in Figure 9. The relative expression level of *ICL1* was reduced by 1.3-fold and 1.5-fold respectively, in the *Δfas1-1* and *Δfas1-2* mutants grown on CM medium. *ICL1* was reduced by more than 2.3-fold and 2.8-fold respectively, in the *Δfas1-1* and *Δfas1-2* mutants (Figure 9) grown on MM supplemented with 50 mM olive oil.

*ICL1* is associated with peroxisomal biogenesis and is involved in the first step of the glyoxylate cycle that encodes isocitrate lyase, which catalyzes the conversion of isocitrate to malate that is responsible for melanin biosynthesis [14,19]. Research shows that *ICL1* gene expression is elevated in the development of appressorium and cuticle penetration and is required for virulence of *M. oryzae* [29]. Production of ATP involving β-oxidation of fatty acids and production of acetyl-CoA is channeled to glycoxylate cycle, which produces glyoxylate through the isocitrate lyase (*ICL*) gene [29]. Therefore, it is possible that in the FAS1 mutants, the ICL1 activity is interrupted, resulting in affected melanin synthesis, pathogenesis and lipid biosynthesis. Results show a higher transcript level of *ICL1* in glucose compared to olive oil, as glucose is the main source of C in the glyoxylate cycle (Figure 9). 

The *PEX6* gene is involved in the glyoxylate cycle that is expressed in the presence of lipid. The relative expression level of *PEX6,* however, increased nearly 4.0-fold in the *Δfas1-1* and 1.4-fold in the *Δfas1-2* respectively, on olive oil medium compared to the wildtype in the same medium (Figure 9). *PEX6* encodes a protein related to peroxisomal biogenesis and is essential for mobilization and degradation of lipid droplets before conidiophore development [14,16,76]. Therefore, peroxisomal biogenesis activity was induced in *Δfas1-1* and *Δfas1-2* mutants compared to wildtype. This is largely contributed by the mutants’ ability to utilize all fatty acids in this study (Figure 8, Supplementary Figures S3 and S4). The *PEX6* gene pathway’s upregulation is indicative of its influence on lipid production and degradation processes on 7–19 C (olive oil) compared to 1–6 C (glucose). The utilization of olive oil induced the upstream lipid biosynthetic pathway, where the *PEX6* gene enters the pathway [76]. This indicates that *FAS1* plays an important role in peroxisomal biogenesis and is essential for mobilization and degradation of lipid droplets, as expression is induced in the presence of 7–19 C fatty acid (olive oil) (Figure 8 and Figure 9). 

## 3. Materials and Methods 

### 3.1. Fungal Strain Culture Condition 

*Magnaporthe oryzae* S6 strain is a Malaysian isolate that was isolated at MADA plantation in Kedah, Malaysia. The isolated fungal strain was obtained from the Plant Genetic Laboratory, Universiti Kebangsaan, Malaysia, and was distributed from a single stock [89]. Cultures were maintained on prune agar with ampicillin antibiotic (50 µg/mL) as stock cultures at 26 °C under dark condition. 

### 3.2. Fungal Genomic DNA Extraction

DNA was extracted using the CTAB (Hexadecyltrimethylammonium bromide) (Supplementary Table S8) extraction method from mycelia with some modification, as mentioned below. Briefly, cell walls of fungal mycelia were broken down by grinding with liquid nitrogen. Then, the extraction buffer is preheated to 65 °C before adding and incubated for 30 min with shaking. Purification was conducted via phenol:chloroform:isoamyl alcohol (25:24:1) which was maintained at pH 8, with incubation for 30 min, followed by precipitation with isopropanol. Samples were centrifuged and the pellets were collected and resuspended in nuclease-free water.

### 3.3. Identification and Sequencing of FAS1 Gene

The extracted DNA samples was used to amplify, purify and sequence the putative *FAS1* gene of *M. oryzae* S6 (MT787294) using the primer walking method. 

### 3.4. Identification and Amplification of Flanking Region Fatty Acid Synthase 

The genome database of *Magnaporthe oryzae* 70–15 was retrieved from the Broad Institute (Massachusetts Institute of Technology, Cambridge, MA, USA) (www.broad.mit.edu/annotation/fungi/). The fatty acid synthase beta subunit dehydratase (*FAS1*) gene’s 5′ and 3′ flanking regions were amplified with restriction sites using Polymerase Chain Reaction (PCR). PCR was performed using 25 µL reactions containing 12.5 µL GoTaq^®^ Green Master Mix (Promega Corporation, Madison, WI, USA), 2X, 2 µL of 10 pM of each primer, genomic DNA (50 ng) and conditions consisting of one cycle of denaturation at 95 °C for 10 min, followed by 35 cycles of 94 °C for 30 s and 72 °C for 1 min, and a final extension at 72 °C for 10 min. 

### 3.5. Homologous Double Crossover Method 

The gene disruption cassette was constructed using the plasmid pN1389, with hygromycin-resistant gene cassette driven by the *Aspergillus niger* glucoamylase promoter [59,90]. PCR-amplified fragments were ligated into the pGEMT-Easy vector and transformed into *E. coli* JM109. Approximately 1000 bp at the 5′ region of the gene was amplified using the primers FAS1pN5 Forward/FAS1pN5 Reverse (Supplementary Table S1), containing *Kpn*I and *Bam*HI sites, respectively. The 3′ region of the gene which is approximately 900 bp was amplified using FAS1pN3 Forward/FAS1pN3 Reverse (Supplementary Table S1) containing *Sda*I and *Sph*I, respectively. Ligation of the 5′ and 3′ flanking regions of FAS1 was digested and ligated into pN1389 to form pN1389-FAS1 with the help of restriction enzymes [60].

### 3.6. Fungal Transformation and Screening

Fungal spheroplasts were generated using Glucanex as described previously [91]. Purified fungal protoplasts (10^6^ to 10^7^ per mL) were transformed using 10 µg of plasmid pN1389-FAS1 via Polyethylene Glycol (PEG)-mediated spheroplasts transformation grown on underlayer Osmotic Complete Medium (OCM) agar with hygromycin (200 µg/mL). The putative transformants isolated from the OCM agar were transferred to CM with hygromycin (200 µg/mL). The putative *fas1* mutant auxotrophs were subjected to PCR verification via HYH Forward/HYH Reverse (Supplementary Table S1) primers designed to amplify the hygromycin gene.

### 3.7. Protein Isolation, Concentration Determination and SDS Analysis

The mycelia were filtered and protein was extracted using phosphate-buffered saline and protein concentration was estimated from measurements of protein culture filtrates performed using Bradford reagent [92]. Protein concentration was estimated using Bradford reagent, 1 µL of protein sample and 49 µL of NaCl (0.15 M), to a final concentration of 0.147 M and 500 µL of Bradford Reagent [92]. Ten-fold dilutions were used to generate a standard curve. The concentration was determined based on the absorbance at 280 nm. The proteins were mixed with 2X sample buffer (Supplementary Table S9) and analyzed by SDS-PAGE using the Mini-PROTEAN system (Bio-Rad Laboratories Inc., Hercules, CA, USA), PAGEr^®^ Precast Protein Gels (Lonza, Walkersville, MD, USA)and color pre-stained protein standard with broad range marker (Biolabs Inc, Ipswich, MA, USA), and was later visualized.

### 3.8. Conidiation, Appressorium Formation and Vegetative Growth Assays

Conidia harvested from Δ*fas1* and wildtype strain after 10 days was collected in 5 mL of distilled water, filtered through 3 layers of lens paper and counted, before being re-suspended to a final concentration of 5 × 10^4^ spores per mL. Appressorium formation was observed under light microscope after 10 µL droplets of conidial suspension were placed on hydrophobic plastic coverslips and incubated under humid condition at a temperature of 26 °C without light for 4 h [16]. Vegetative growth was assessed by measurement of colony diameter of plate culture after 10 days of incubation on complete medium and minimum medium agar at 26 °C. A cube size agar with mycelium and developed spores was removed from the oat agar plate for SEM analysis.

### 3.9. Fatty Acid Synthase Beta Subunit Dehydratase Gene (FAS1) Assay Using ELISA Kit 

Enzyme-Linked Immunosorbent Assay Kit for Fatty Acid Synthase (FASN) (Cloud Clone Corporation, Katy, TX, USA) was used in this assay. This kit is a sandwich enzyme immunoassay for in vitro quantitative measurement of FASN in mouse tissue, homogenates, cell lysate and other biological fluids. The microplate provided in this kit has been pre-coated with an antibody specific to FASN only. The protein samples extracted from fungus cultivated in complete and minimum media and kit components was brought to room temperature (18–25 °C) before use. All reagents, samples and standards were prepared with accurate dilution. Details of reactions and method for analysis is as recommended by the manufacturer.

### 3.10. FAS1 Gene Expression Analysis

First-strand cDNA was synthesized using QuantiNova Reverse Transcription Kit from 2 µg RNA. Quantitative RT-PCR was used to characterize *PEX6* and *ICL1* gene expression. Each qRT-PCR mixture (final volume 20 µL), which contained QuantiNova SYBR Green PCR (Qiagen, Hilden, NRW, German), forward and reverse primer and cDNA template was subjected to PCR in the iCycler iQ5 Real-Time PCR Detection System (Bio-Rad, Hercules, CA, USA). *EF1α* is elongation factor 1-alpha encoding gene (MGG_03641.5) and is used as a control gene and wildtype strain S6 is used as a calibrator [93]. PCR conditions were 94 °C for 2 min, 95 °C for 5 min and 72 °C for 10 seconds. The relative quantification of the transcripts was calculated by the 2^−*ΔΔ*Ct^ method and values were obtained from three independent biological experiments with three technical replicates for each independent experiment [94].

### 3.11. Multiple Sequence Alignment and Phylogenetic Tree

The results of the multiple alignment data generated were used to build the phylogenetic relationship between our isolate and those downloaded from NCBI. In this study, a Maximum Likelihood analysis with bootstrap value 1000 was conducted on 105 sequences of amino acids of FAS1 protein (Figure 3). The MEGA X program (MEGA, University Park, PA, USA) was used to derive this tree [95].

### 3.12. Statistic Analysis Calculation

Sample testing was performed using an ANOVA (Analysis of Variance) via GraphPad Prism v8.4.3 (GraphPad Software, San Diego, CA, USA). to calculate the significant value of the experiment. The significance of treatments was determined by the magnitude of *p*-value [96].

## 4. Conclusions

In brief, the morphological phenotypes of *Δfas1-1* and *Δfas1-2* mutants were similar to each other, including abnormal spore appearance with less septa, asymmetrical teardrop-shaped spores, impaired lipid distribution and less dense mycelia. Taken together, *FAS1* is a crucial gene for development of mycelium structure in terms of cell wall composition, lipid synthesis and translocation. *FAS1* is able to utilize 1 to 19 C fatty acids as a sole carbon source and is required for lipid utilization and pathogenesis. *FAS1* may also be involved in sporulation, conidium morphology and the melanin biosynthesis pathway [12,85,86,87]. Further, biochemical and gene expression studies suggest that the *FAS1* gene contributes to turgor pressure for appressorium formation, specifically in generating glycerol and degradation of lipid bodies. Relative expression levels of the *FAS1* gene using genes associated with melanin production and the lipid pathway concluded that all machinery related to the fungal pathogenicity may be affected with the deletion of *FAS1.* However, only pathogenicity testing on resistant and susceptible rice plant lines will conclusively determine the effect on virulence and pathogenicity of these mutants. *ICL1* and *PEX* genes were selected for use in comparing the wildtype and mutants, as these genes were closely related in the melanin, glyoxylate, PTS1, PTS2 and lipid biosynthesis pathways, which are crucial in the degradation of lipid and plays a key role in fungal development, the infection process and pathogenicity. 

Further, it will also be of interest to study the functions of fatty acid degradation genes that may contribute to appressorium turgor pressure and why the contribution has evolved into pathogenic capability in *M. oryzae.* In conclusion, this study has only elaborated on the roles of *FAS1* in *Magnaporthe oryzae S6.* The next challenge will be to determine how the other three lipid biosynthesis genes (fatty acid synthase subunit alpha (FAS2), Acetyl- CoA carboxylase [27] and 3-oxoacyl-{acyl-carrier-protein} synthase II (FAbB)) recognized through KEGG will collectively influence fungal pathogenicity of *M. oryzae*. Further, we could also look into the role of alternative pathways in regulating the function of FAS1.

## Figures and Tables

**Table 1:** Fatty acid synthase activity in wildtype and mutant *M. oryzae* strain S6 of two biological replicates. Mycelium of wildtype *M. oryzae* strain S6, *Δfas1-1* and *Δfas1-2* were grown in CM for 24 h and then transferred to MM containing 50 mM of olive oil for 24 h before harvesting.

Media	CM (ng/mL)	Olive Oil (ng/mL)
Wildtype	0.363	0.000
*fas1-1*	0.000	0.000
*fas1-2*	0.000	0.000

## Supplementary Materials

The following are available online at https://www.mdpi.com/1422-0067/21/19/7224/s1, Figure S1: The nucleotide and protein sequence of the FAS1 gene of *Magnaporthe oryzae* 70-15, Figure S2: Multiple sequence alignment of 5 homologous of Ascomycota, Figure S3: Geographical distribution of ascomycetes, which are closely related to *Magnaporthe oryzae* S6, Figure S4: SDS-PAGE, Table S1: Primers used in this study, Table S2: Table to show ascomycetes fungus strain, phylum, substrate, host and country which are closely related to *Magnaporthe oryzae* S6 Malaysian strain, Table S3: Diameter measurement of wildtype, *Δfas1-1* and *Δfas1-2* mutants of three biological replicates of each different carbon sources, 10 days culture under on complete media agar plates at temperature 26 °C, Table S4: Mycelial dry weight wildtype, *Δfas1-1* and *Δfas1-2* mutants of three biological replicates in each different carbon sources that were grown for 10 days under (12 hours day light, 12 hours dark) on complete media agar plates at temperature 26 °C, Table S5: Number of conidia harvested from a 9 cm oatmeal agar at day 10 after incubation at 26°C, Table S6: Size measurement of conidia length and width of 30 conidia in micrometre wildtype, *Δfas1-1* and *Δfas1-2* mutants that were grown for 10 days under (12 hours day light, 12 hours dark) on complete media agar plates at temperature 26 °C, Table S7: Conidia length and conidia width measurement of wildtype, *Δfas1-1* and *Δfas1-2* mutants of 10 days under on prune and oat agar plates at temperature 26 °C, Table S8: DNA extraction buffer, CTAB (Hexacetyltrimethylammonium bromide) buffer recipe, Table S9: Sodium dodecyl sulfate polyacrylamide gel electrophoresis (SDS-PAGE) sample buffer recipe.
