# Supplementary material for: Fatty Acid Synthase Beta Dehydratase in the Lipid Biosynthesis Pathway Is Required for Conidiogenesis, Pigmentation and Appressorium Formation in Magnaporthe oryzae S6
Source: Int J Mol Sci. 2020 Sep 30;21(19):7224. doi: 10.3390/ijms21197224 (PMC7582888; doi:10.3390/ijms21197224)
Supplement: Supplementary file 1 [file ijms-21-07224-s001.pdf]

Table S1. Primers used in this study

Primer	Sequence 5′-3′
FAS1pN5 Forward	GGTACCGAACGACACCCTAAAGAATTC
FAS1pN5 Reverse	GGATCCATTGTGGTTTGTCACGGTCCG
FAS1pN3 Forward	CCTGCAGGAAGAGTAAGATGGCGTCGTGA
FAS1pN3 Reverse	GCATGCCGCTCCTTTCCCAACCAAATT
FAS1A Forward	ACACCTACGCGAGACAGGAGA
FAS1A Reverse	CGGTGGCTTGTC AGCATCTATG
HYH Forward	CATTACACCTCAGCCATGG
HYH Reverse	GGGCCCATGTCAACAAGAAT
PEX6 Forward	AAATTCACACTCCACCCTTCA
PEX6 Reverse	GGCACTCACACTGGGAATC
ICL1 Forward	GTGTACCCCGAGCAAAAACT
ICL1 Reverse	ACCCGTCACCATCTTCTGC
EF1α Forward	TCAGAAGCATCATTGCTCCG
EF1α Reverse	GATCATGAAGTGCGATGTCGA

(A) FAS1 nucleotide sequence

```
atgtacggcaccggtaccggccctcaaacgggcgtttctacgccgcggtcttcggcctcg 60
 M  Y  G  T  G  T  G  P  Q  T  G  V  S  T  P  R  S  S  A  S
ctccgcccattgaacctcacacacggctccctcgagacgtcgttcctaatacccacaaat 120
 L  R  P  L  N  L  T  H  G  S  L  E  T  S  F  L  I  P  T  N
ctgcacttccatgcctctcagcttaaagatcggttcagtgcaagtttgcctgctcctacg 180
 L  H  F  H  A  S  Q  L  K  D  R  F  S  A  S  L  P  A  P  T
gatgagctggcacaagatgacgagccttcgtcggttcccgagctcgttgcgaggtacatg 240
 D  E  L  A  Q  D  D  E  P  S  S  V  P  E  L  V  A  R  Y  M
ggattcgtcgcccgcgaagttgaggaggagggtgacgatgctcaaggctcctacgaggaa 300
 G  F  V  A  R  E  V  E  E  E  G  D  D  A  Q  G  S  Y  E  E
gtccttaagcttcttcttaacgagtttgagcgtgtcttcctcaggggcaacgaagttcac 360
 V  L  K  L  L  L  N  E  F  E  R  V  F  L  R  G  N  E  V  H
gctctagcagccactctgcctggaattgacactaagaagctagaagtcattcgcagctat 420
 A  L  A  A  T  L  P  G  I  D  T  K  K  L  E  V  I  R  S  Y
tttgccaccagatacgtcttgaaccgagccatcaagccgcatgcgtctgccctcttcagg 480
 F  A  T  R  Y  V  L  N  R  A  I  K  P  H  A  S  A  L  F  R
gcgtcggctgaggacaaggccaagatctacaccatattcggaggacagggcaacattgag 540
 A  S  A  E  D  K  A  K  I  Y  T  I  F  G  G  Q  G  N  I  E
gagtactttgaggagctccgtgagctccacacaacctaccgtgtttttatagaggagctc 600
 E  Y  F  E  E  L  R  E  L  H  T  T  Y  R  V  F  I  E  E  L
atcactacgtcggcagagctgcttcaaaacctgtccaaggatccatcagcggagaagctg 660
 I  T  T  S  A  E  L  L  Q  N  L  S  K  D  P  S  A  E  K  L
ttttccaaaggcctcgatgtcatgtcatggcttcacaggcctgaggccacaccggacatt 720
 F  S  K  G  L  D  V  M  S  W  L  H  R  P  E  A  T  P  D  I
gattacctggtgtctgcacccgttagctttcctctaattggtttggtccagcttgctcac 780
 D  Y  L  V  S  A  P  V  S  F  P  L  I  G  L  V  Q  L  A  H
tatgaggtgacctgtaaagttttgggcgttcccccaagcgctctccgcgagaggttcagc 840
 Y  E  V  T  C  K  V  L  G  V  P  P  S  A  L  R  E  R  F  S
```

ggcgccactggacattcccaaggcattgttcttgcagctgccacagctgctgctgatacc 900
G A T G H S Q G I V L A A A T A A A D T
tggaaagaatggcccactattgtcaagaagaccttgaccatcttgttctggattggagcg 960
W K E W P T I V K K T L T I L F W I G A
cgcagccagcagacctttccccgcacttcaatcacgccgtcgattcttcaagattctgtc 1020
R S Q Q T F P R T S I T P S I L Q D S V
gacaatggagagggaatccccacccctatgcttagcatcagggatcttccccaagccgag 1080
D N G E G I P T P M L S I R D L P Q A E
gtacagaagcacattgacaagaccaacgagtaccttcccgcggatcgccatatctccatt 1140
V Q K H I D K T N E Y L P A D R H I S I
tctctcataaacagtccccgcaacatggttgttacaggacctccgatttctctctacggt 1200
S L I N S P R N M V V T G P P I S L Y G
ctcaacctccagctcagaaaagtcaaggccccaaccggtcttgaccagaacagaataccg 1260
L N L Q L R K V K A P T G L D Q N R I P
tacactcagcgaaaggttcgctttgtgaacaggttcttacctatcaccgcgccatttcac 1320
Y T Q R K V R F V N R F L P I T A P F H
agcaagtacttggctgaagccacctctatgattgataacgacttgcagtacactcacttc 1380
S K Y L A E A T S M I D N D L Q Y T H F
gaagtgaaggacctcaagatccccgtctttgacactcacactggcaaagacattcgagag 1440
E V K D L K I P V F D T H T G K D I R E
gaggttaagggtaacatcgttcccaccctcatccgactcatcacccgggacccggtccac 1500
E V K G N I V P T L I R L I T R D P V H
tgggagaaggcaactgtcttccccggcgcaactcatgttcttgatttcggcccaggtgga 1560
W E K A T V F P G A T H V L D F G P G G
atctccggattgggaattctcaccagccgtaacaaggatggtactggagtacgcgtcatc 1620
I S G L G I L T S R N K D G T G V R V I
ctagctggctctgttgctggaactgtgacagaggtgggttacaagccagagctttttgac 1680
L A G S V A G T V T E V G Y K P E L F D
cgggatgaggaacacgctgtgaagtacgctattgattgggtcaaggagtacggtcccaag 1740
R D E E H A V K Y A I D W V K E Y G P K
ttgatcaagactgccactggccgcacctacgttgataccaagatgagtaggctgcttgga 1800
L I K T A T G R T Y V D T K M S R L L G
cttcctcctgtcatggtagcaggtatgaccccgtgcactgtcaaatgggactttgttgcg 1860
L P P V M V A G M T P C T V K W D F V A
gcaaccatgaacgctggttatcacatcgagctggcgggaggcggttactttgagcctagg 1920
A T M N A G Y H I E L A G G G Y F E P R
atgatgaccgatgcccttgtcaagattgagaaggcgattcctgctggccgcggtatcagc 1980
M M T D A L V K I E K A I P A G R G I S
gtcaaccttatctacgtcaacccccgcgcgatgcagtggcagataccgttgattgggcgt 2040
V N L I Y V N P R A M Q W Q I P L I G R
cttcgatctcaaggcgttcccatcgagggtctcactatcggtgctggtgtgccttcgatc 2100
L R S Q G V P I E G L T I G A G V P S I
gaggttgccaacgaatacatccaaactctgggtctcaagcacatttctttcaagccggga 2160
E V A N E Y I Q T L G L K H I S F K P G
tcgacagacgctattcaagctgtcatcaacattgccaatgccaaccccacattccccgtc 2220
S T D A I Q A V I N I A N A N P T F P V
atgctgcagtggactggaggtcgtggtggtggacaccactcgttcgaggacttccaccag 2280
M L Q W T G G R G G G H H S F E D F H Q
cctgtgctgctcatgtacaacaggattcgcaagtgtgacaacctgatcctgatcgcaggc 2340
P V L L M Y N R I R K C D N L I L I A G
agcggcttcggtggtgccgaggacacttatccttacatcactggagagtggtccagaaag 2400
S G F G G A E D T Y P Y I T G E W S R K
ttcggttatcccccccatgcctttcgatggttgtctgtttggaagtcgcgtcatggttgcc 2460
F G Y P P M P F D G C L F G S R V M V A
aaggaggcgcacactgccccggcagccaagcaagccatcgtcgatgcacctggtgttgac 2520
K E A H T A P A A K Q A I V D A P G V D
gatgacgagtgggagaaaacttacaatggcgttgcaggtggcatcacgaccgttctgtcg 2580
D D E W E K T Y N G V A G G I T T V L S
gagatgggagagccaatccacaagattgccacgcgtggtgtcaagttctggtccgagatg 2640
E M G E P I H K I A T R G V K F W S E M
gaccagaagatcttcagcttgcccaaagagaagcgtgtcccggagctcaagaagatgcgc 2700

D Q K I F S L P K E K R V P E L K K M R
agctacataatcgagaagttaaacaaggacttccacaaagtttggttcggcaagaacaag 2760
S Y I I E K L N K D F H K V W F G K N K
gccggacaggccgttgaccttgaggacatgacatacgccgaggtagtccgtcgcatggtc 2820
A G Q A V D L E D M T Y A E V V R R M V
gagctcatgtatgtcaagcatgagtctcgctggattgaccccagcttcatgcgtttgact 2880
E L M Y V K H E S R W I D P S F M R L T
ggtgacttcatccaccgagtcgaggagcggtttgttcagagctctgggcagccttctctt 2940
G D F I H R V E E R F V Q S S G Q P S L
ctccaacactacacagatcttacacaacccttcgagactattgaaaagatcctggcacac 3000
L Q H Y T D L T Q P F E T I E K I L A H
tatcctcaggctgaggatcagcttatcaacgcccaggatgtccagcacttccttcaactc 3060
Y P Q A E D Q L I N A Q D V Q H F L Q L
tgcatgaggccaatccagaagcctgttgttttcgtgccagctctggacgacaattttgac 3120
C M R P I Q K P V V F V P A L D D N F D
ttctacttcaaaaaagactcactgtggcagtccgaggatctccaggccgtcatggaccag 3180
F Y F K K D S L W Q S E D L Q A V M D Q
gacgtccagcgtacttgtattttgcagggaccaatggctgttcgctattctactgtggtc 3240
D V Q R T C I L Q G P M A V R Y S T V V
gacgagcctatccagagcatccttgatggcgttcatgaagggcacattgccggtctcagc 3300
D E P I Q S I L D G V H E G H I A G L S
aaagatttgtaccaaggaaagacatcaaccctcccttcgatcgagtacttcggaggaaag 3360
K D L Y Q G K T S T L P S I E Y F G G K
ctcatggagcgtgaattccccgatgttgactcgattgatggtttgagtgtttccgaggaa 3420
L M E R E F P D V D S I D G L S V S E E
agaggtgttagagacatcttccgcatctcgctgtccccgtctcagaccatgccaagcacg 3480
R G V R D I F R I S L S P S Q T M P S T
gatcattggttgtcactcattgccggaccgacccgctcatggcgccacgccttgttgatg 3540
D H W L S L I A G P T R S W R H A L L M
tcggaggtgattgtccaaggcacaaagttccagagcaacccgatgaggcgtattttgcc 3600
S E V I V Q G T K F Q S N P M R R I F A
ccttgccgcggtctttttgttgaagttcgctaccccaaggatccagcatccacacaaatc 3660
P C R G L F V E V R Y P K D P A S T Q I
attgttagggagcaacccagacacaaccactacgtcgatgttttggttgcccgtctctct 3720
I V R E Q P R H N H Y V D V L V A R L S
Ggcaagaatgagatcattgtagagctgatcaaggacaccactgcattgggtaagccggtt 3780
G K N E I I V E L I K D T T A L G K P V
Tctctgcctctcaagttcatctataagcccgaggctggatacgctcctctgcacgaagtc 3840
S L P L K F I Y K P E A G Y A P L H E V
Atggatgaccgcaatgaccgcatcaaggagttttactggcgagcttggtttggcgatgag 3900
M D D R N D R I K E F Y W R A W F G D E
Aagcttgagctggatgccaacgtcgacagtgcctttgacggaggcaaggatgtcgtgaca 3960
K L E L D A N V D S A F D G G K D V V T
Agcgaagcgatcaacgactttgtccatgctgtcggcaacacgggcgaagccttcgttgac 4020
S E A I N D F V H A V G N T G E A F V D
Cggcctggaaaggtcatgtatgcgcccatggacttcgccatcgttgtcggctggaaggca 4080
R P G K V M Y A P M D F A I V V G W K A
Ataaccaagcccattttccctcgcaagatcgatggtgacctcctcaaccttgtccacctt 4140
I T K P I F P R K I D G D L L N L V H L
Tccaaccagttccgcatgaagccaggtgctgagcctctcaagaagggagatgaggtttct 4200
S N Q F R M K P G A E P L K K G D E V S
Acagtttcacgagtcaacgccgttgtcattcaagaatctggaaagatggtcgaggtctgc 4260
T V S R V N A V V I Q E S G K M V E V C
Ggtaccatcacccgcgatggtgaaccagtcatggaggtgacatcccagtttttgtaccgg 4320
G T I T R D G E P V M E V T S Q F L Y R
Ggaacctacaccgatttcgagaacacattccagagcaagatcgagacaccaatgcagatc 4380
G T Y T D F E N T F Q S K I E T P M Q I
Cacctcgcgacttccaaggacgtcgccgttctacggtcgaaggaatggttcaccctcgaa 4440
H L A T S K D V A V L R S K E W F T L E
Gacaacgttaacatggacttgctggggcagacacttatcttccgtctccagtctcttgtc 4500
D N V N M D L L G Q T L I F R L Q S L V

Cgtttcaagaacaccaccgtgttcagcagcgtgcagactcgcggtgaggtacttgtagag 4560
R F K N T T V F S S V Q T R G E V L V E
Ctgcctacaaaggagatcgtccaagtcggcagtgtcgagtatgaagctggtacctcgcac 4620
L P T K E I V Q V G S V E Y E A G T S H
Ggaaaccccgtgattgatttcttggagcgaaacggttcttcgatcgagcagccaatcaac 4680
G N P V I D F L E R N G S S I E Q P I N
Tttgagaacccgattccccttagtggcaagactcccctccagcttcgggcccccgcctcg 4740
F E N P I P L S G K T P L Q L R A P A S
Aacgagacatacgccagagtgtctggcgactacaaccccattcacgtttctcgggttttt 4800
N E T Y A R V S G D Y N P I H V S R V F
Gccaactatgccgatctgccgggcaccatcacccatggcatgtactctagtgccgcggtg 4860
A N Y A D L P G T I T H G M Y S S A A V
Cgtagcctggtcgagacttgggcagctgaaaatgacaacagcagagtcaggagcttccac 4920
R S L V E T W A A E N D N S R V R S F H
Gcatcattggtcggcatggtgctgcccaacgatgacttgctggtcaaattgcagcatgtt 4980
A S L V G M V L P N D D L L V K L Q H V
Ggtatggttgccggacgcaagatcatcaaggttgaggtcatcaacaaggagactgaggac 5040
G M V A G R K I I K V E V I N K E T E D
Aaggtgctgttgggagaggccgaggttgagcagcccgtgaccgcgtacgtgttcactgga 5100
K V L L G E A E V E Q P V T A Y V F T G
Caaggttcgcaagagcaaggtatgggcatggagctgtacgaaagcagcccggttgccaag 5160
Q G S Q E Q G M G M E L Y E S S P V A K
Gaagtttgggaccgcgcagacaagtacttgatggacacatatggattcgcgatcacaaat 5220
E V W D R A D K Y L M D T Y G F A I T N
Attgtcaagaacaaccccaaggagctcaccattcactttggaggccctcgtggcaaggct 5280
I V K N N P K E L T I H F G G P R G K A
Attcgcgccaactacatggccatgaccttcgagactgttgctgcggacggaaccatcaaa 5340
I R A N Y M A M T F E T V A A D G T I K
Tctgagcgcatcttcaaggatatcaacgagaggacaacttcatacacttaccggtctcct 5400
S E R I F K D I N E R T T S Y T Y R S P
Acaggcctgctctcagcgacacagttcactcaacccgccctgacactgatggagaaggca 5460
T G L L S A T Q F T Q P A L T L M E K A
Agcttcgaagacatgaaggctaagggactggtccctcgcgacagtacttttgccggtcat 5520
S F E D M K A K G L V P R D S T F A G H
Tccttgggtgaatactcggctctcgctgccctggctgatgtcatgcccattgagagtttg 5580
S L G E Y S A L A A L A D V M P I E S L
Gtctctgtcgttttctaccgtggtctcacgatgcaggtcgctgtggagcgtgacgccagt 5640
V S V V F Y R G L T M Q V A V E R D A S
Ggaaggtccaactactcaatgtgcgccgtcaacccgagccgtatctccaagaccttcaac 5700
G R S N Y S M C A V N P S R I S K T F N
Gaggaggctctccgcttcgtcgtcggcaacatcgccgagggaacgggctggttgctcgag 5760
E E A L R F V V G N I A E G T G W L L E
Atcgtcaactacaacatcgcaaacatgcaatacgtttgcgctggagatctccgggctctg 5820
I V N Y N I A N M Q Y V C A G D L R A L
Gatacgcttaccaatgtgctcaactacctcaaggcacagaagatcgacattgaggctatg 5880
D T L T N V L N Y L K A Q K I D I E A M
Aaggctgccatgacagtcgaggacgtcaagcagcacctgcaggagatcatcaaggcgtcc 5940
K A A M T V E D V K Q H L Q E I I K A S
Gccgagaagacggaggccaagcccaagcccttggagcttgagaggggctttgcaaccatc 6000
A E K T E A K P K P L E L E R G F A T I
Ccgctgcgtggaatcgacgtgcctttccattcgacctttttgcgcagcggtgtaaagccg 6060
P L R G I D V P F H S T F L R S G V K P
Tttaggtcattcctgcttaagaagatcaacaagacaaccattgacccctcaaaactgctc 6120
F R S F L L K K I N K T T I D P S K L L
Ggaaagtacattcctaacgtgaccgcgaagcctttcgcactcaccaaggagtacttcgag 6180
G K Y I P N V T A K P F A L T K E Y F E
Gatgtctataggttgaccaactcgcctcgcattgcttcgatcttggccaactgggagcaa 6240
D V Y R L T N S P R I A S I L A N W E Q
Taccagaacgagaacgcggttccgtccaccatcggccttggcgccaacggtgcgggcgat 6300
Y Q N E N A V P S T I G L G A N G A G D
Gccaacggcgagaccaccgagaatggcgtggaagaagcgaacggcgccaacggagtccac 6360

```
 A  N  G  E  T  T  E  N  G  V  E  E  A  N  G  A  N  G  V  H
tag
 *
```

(B) FAS1 protein sequence

```
MYGTGTGPQTGVSTPRSSASLRPLNLTHGSLETSFLIPTNLHFHASQLKDRFSASLPAPTDELAQDDEPS 70
SVPELVARYMGFVAREVEEEGDDAQGSYEEVLKLLLNEFERVFLRGNEVHALAATLPGIDTKKLEVIRSY 140
FATRYVLNRAIKPHASALFRASAEDKAKIYTIFGGQGNIEEYFEELRELHTTYRVFIEELITTSAELLQN 210
LSKDPSAEKLFSKGLDVMSWLHRPEATPDIDYLVSAPVSFPLIGLVQLAHYEVTCKVLGVPPSALRERFS 280
GATGHSQGIVLAAATAAADTWKEWPTIVKKTLTILFWIGARSQQTFPRTSITPSILQDSVDNGEGIPTPM 350
LSIRDLPQAEVQKHIDKTNEYLPADRHISISLINSPRNMVVTGPPISLYGLNLQLRKVKAPTGLDQNRIP 420
YTQRKVRFVNRFLPITAPFHSKYLAEATSMIDNDLQYTHFEVKDLKIPVFDTHTGKDIREEVKGNIVPTL 490
IRLITRDPVHWEKATVFPGATHVLDFGPGGISGLGILTSRNKDGTGVRVILAGSVAGTVTEVGYKPELFD 560
RDEEHAVKYAIDWVKEYGPKLIKTATGRTYVDTKMSRLLGLPPVMVAGMTPCTVKWDFVAATMNAGYHIE 630
LAGGGYFEPRMMTDALVKIEKAIPAGRGISVNLIYVNPRAMQWQIPLIGRLRSQGVPIEGLTIGAGVPSI 700
EVANEYIQTLGLKHISFKPGSTDAIQAVINIANANPTFPVMLQWTGGRGGGHHSFEDFHQPVLLMYNRIR 770
KCDNLILIAGSGFGGAEDTYPYITGEWSRKFGYPPMPFDGCLFGSRVMVAKEAHTAPAAKQAIVDAPGVD 840
DDEWEKTYNGVAGGITTVLSEMGEPIHKIATRGVKFWSEMDQKIFSLPKEKRVPELKKMRSYIIEKLNKD 910
FHKVWFGKNKAGQAVDLEDMTYAEVVRRMVELMYVKHESRWIDPSFMRLTGDFIHRVEERFVQSSGQPSL 980
LQHYTDLTQPFETIEKILAHYPQAEDQLINAQDVQHFLQLCMRPIQKPVVFVPALDDNFDFYFKKDSLWQ 1050
SEDLQAVMDQDVQRTCILQGPMAVRYSTVVDEPIQSILDGVHEGHIAGLSKDLYQGKTSTLPSIEYFGGK 1120
LMEREFPDVDSIDGLSVSEERGVRDIFRISLSPSQTMPSTDHWLSLIAGPTRSWRHALLMSEVIVQGTKF 1190
QSNPMRRIFAPCRGLFVEVRYPKDPASTQIIVREQPRHNHYVDVLVARLSGKNEIIVELIKDTTALGKPV 1260
SLPLKFIYKPEAGYAPLHEVMDDRNDRIKEFYWRAWFGDEKLELDANVDSAFDGGKDVVTSEAINDFVHA 1330
VGNTGEAFVDRPGKVMYAPMDFAIVVGWKAITKPIFPRKIDGDLLNLVHLSNQFRMKPGAEPLKKGDEVS 1400
TVSRVNAVVIQESGKMVEVCGTITRDGEPVMEVTSQFLYRGTYTDFENTFQSKIETPMQIHLATSKDVAV 1470
LRSKEWFTLEDNVNMDLLGQTLIFRLQSLVRFKNTTVFSSVQTRGEVLVELPTKEIVQVGSVEYEAGTSH 1540
GNPVIDFLERNGSSIEQPINFENPIPLSGKTPLQLRAPASNETYARVSGDYNPIHVSRVFANYADLPGTI 1610
THGMYSSAAVRSLVETWAAENDNSRVRSFHASLVGMVLPNDDLLVKLQHVGMVAGRKIIKVEVINKETED 1680
KVLLGEAEVEQPVTAYVFTGQGSQEQGMGMELYESSPVAKEVWDRADKYLMDTYGFAITNIVKNNPKELT 1750
IHFGGPRGKAIRANYMAMTFETVAADGTIKSERIFKDINERTTSYTYRSPTGLLSATQFTQPALTLMEKA 1820
SFEDMKAKGLVPRDSTFAGHSLGEYSALAALADVMPIESLVSVVFYRGLTMQVAVERDASGRSNYSMCAV 1890
NPSRISKTFNEEALRFVVGNIAEGTGWLLEIVNYNIANMQYVCAGDLRALDTLTNVLNYLKAQKIDIEAM 1960
KAAMTVEDVKQHLQEIIKASAEKTEAKPKPLELERGFATIPLRGIDVPFHSTFLRSGVKPFRSFLLKKIN 2030
KTTIDPSKLLGKYIPNVTAKPFALTKEYFEDVYRLTNSPRIASILANWEQYQNENAVPSTIGLGANGAGD 2100
ANGETTENGVEEANGANGVH
```

Figure S1. A. The nucleotide and protein sequence of the FAS1 gene of *Magnaporthe oryzae* 70-15. The sequence shows alignment of nucleotide sequence above and amino acid sequence below FAS1 gene using ExPASy Translate Tool. The asterisk indicates the location of the STOP CODON. B. The translated protein sequence of the FAS1 protein. The sequences were obtained from NCBI.

```
CLUSTAL O(1.2.4) multiple sequence alignment


NP_594370.1         ----------MVEAEQVHQSLRSLVLSYAHFSPSILIPASQYLLAAQLRDEFLSLHPAPS      50
XP_003719712.1      MYGTGTGPQTGVSTPRSSASLRPLNLTHGSLETSFLIPTNLHFHASQLKDRFSASLPAPT      60
XP_962465.1         MYGTGTGAQTGVTTPRSSASLRPLTVSHGSLETSFLIPTGLHFHATQLKERFAAILPAPT      60
NP_984945.2         ---------------MSGITTRSLALGHGALEHTLSVPTGLYLTASQLAASFAKEQVAPT      45
NP_012739.1         ---------------MDAYSTRPLTLSHGSLEHVLLVPTASFFIASQLQEQFNKILPEPT      45
XP_451653.1         ----------------MSSSTRPLSLSHGSIEHVLSIPTSSFFTASQLQDQFHKQLPEAT      44
                                       : * * : :. :.  : :*:  .: *:**   *       :

NP_594370.1         AESVEKEGAELEFEHELHLLAGFLGLIAAK----EEETPGQYTQLLRIITLEFERTFLAG      106
XP_003719712.1      DELAQDDEPS----SVPELVARYMGFVAREVEEEGDDAQGSYEEVLKLLLNEFERVFLRG      116
XP_962465.1         DELAQDDEPS----SIFELVARYMGFIATEVTQGEDDAQGSYEEVLKIILNEFERAFLRG      116
NP_984945.2         EGYASDEEPA----SSAELLAKFVGHVAAQ--A---GAGGEVAAVLELALDEFEASYMCG      96
NP_012739.1         EGFAADDEPT----TPAELVGKFLGYVSSLVEP---SKVGQFDQVLNLCLTEFENCYLEG      98
XP_451653.1         EGFASDDEPT----SSAELLAKFLGYVSSLVDV---TTKGQFDDVLEIALSDFEANYLQG      97
                       . .:          .*:. ::* ::           *.   :*.:   :**  :: *

NP_594370.1         NEVHAVVHSLGL------NIPAQKDVVRFYYHSCALIGQTTKF-HGSALL---DESSVKL      156
XP_003719712.1      NEVHALAATLPGIDT------KKLEVIRSYFATRYVLNRAIKP-HASALFRASAEDKAKI      169
XP_962465.1         NDVHALVSTMEGIDD------KKLEVIRCYYLARSASNRAIKP-YESALFRAAGENAAKI      169
NP_984945.2         EEVHRVAARLLADDEQPTALPKVKELVQHYVYGSVRAQRPLMHR-RPALLESATRGDARV      155
NP_012739.1         NDIHALAAKLLQEND--TTLVKTKELIKNYITARIMAKRPFDKKSNSALFRAVGEGNAQL      156
XP_451653.1         NDVHTLAARLLQSEDNITTLPKVKELIKNYYNARVTAKKPFTA-TDSALFKGVAAGQAKL      156
                    :::* :.  :              :::: *        :        **:     . .::

NP_594370.1         AAIFGGQGY-EDYFDELIELYEVYAPFAAELIQVLSKHLFTLS-QNEQASKVYSKGLNVL      214
XP_003719712.1      YTIFGGQGNIEEYFEELRELHTTYRVFIEELITTSAELLQNLS-KDPSAEKLFSKGLDVM      228
XP_962465.1         FTIFGGQGNIEEYFEELRELHHTYPAFVGELITTSAELLQTLS-SHPDAEKMYPKGLDVL      228
NP_984945.2         VAVFGGQGNTDDYFEELRELHQMYGVLVEDVLDTAAEQLNELLRTTEGAEKMYTQGLDFR      215
NP_012739.1         VAIFGGQGNTDDYFEELRDLYQTYHVLVGDLIKFSAETLSELIRTTLDAEKVFTQGLNIL      216
XP_451653.1         VSIFGGQGNTDDYFEELRELYQTYNCLISDVIKSAAETLGELITTTDDTEKLYTQGFDIL      216
                     ::*****  ::**:** :*:  *  :  :::   :: *  *      :.*:: :*::.

NP_594370.1         DWLAG--ERPERDYLVSAPVSLPLVGLTQLVHFSVTAQILGLNPGELASRFSAASGHSQG      272
XP_003719712.1      SWLHRPEATPDIDYLVSAPVSFPLIGLVQLAHYEVTCKVLGVPPSALRERFSGATGHSQG      288
XP_962465.1         GWLQSPEATPDVDYLISAPVSFPLIGLVQLAHYQVTCKVLGVHPGVLRERISGTTGHSQG      288
NP_984945.2         QWLAQPERTPDNDYLLLIPISCPLIGILQLAHYAVTARIVGITPGELRACMVGVTGHSQG      275
NP_012739.1         EWLENPSNTPDKDYLLSIPISCPLIGVIQLAHYVVTAKLLGFTPGELRSYLKGATGHSQG      276
XP_451653.1         TWLEQPQKTPGTDYLLSIPISCPLIGVIQFVHYTLTARILNFTPGQLRDSLVGATGHSQG      276
                     **      *  ***:  *:* **:*: *:.*: :*.:::.. *. *   : ..:*****

NP_594370.1         IVVAAAVSASTDSASFMENAKVALTTLFWIGVRSQQTFPTTTLPPSVVADSLASSEGNPT      332
XP_003719712.1      IVLAAATAAADTWKEWPTIVKKTLTILFWIGARSQQTFPRTSITPSILQDSVDNGEGIPT      348
XP_962465.1         VVLSAITAAADSWESFEEHSKAALTILFWIGARSQQTFPRTSLAPSMLQDSADNGEGVPT      348
NP_984945.2         LSTAVAVAEADTWESFFCALRKTVSLLFFIGVRCYQVYPKTSLPPSILEDSLENGEGKPS      335
NP_012739.1         LVTAVAIAETDSWESFFVSVRKAITVLFFIGVRCYEAYPNTSLPPSILEDSLENNEGVPS      336
XP_451653.1         LATAVAIAEADSWESYFKAMTKTIKLLFFIGVRCYQVYPKTSLPPSMLEDSVENGEGVPT      336
                    :  :.  : :    .:      ::. **:**.*. ..:* *:: **:: **  ..** *:
```

```
NP_594370.1         PMLAVRDLPIETLNKHIETTNTHLPEDRKVSLSLVNGPRSFVVSGPARSLYGLNLSLRKE     392
XP_003719712.1      PMLSIRDLPQAEVQKHIDKTNEYLPADRHISISLINSPRNMVVTGPPISLYGLNLQLRKV     408
XP_962465.1         PMLSIRDLPQAEVQKHIDQTNAYLPEDQHISIALINSPRNMVVAGPPSSLHGLNLQLRKL     408
NP_984945.2         PMLSVSNLTQEQVQEFVEKTNAHLPSEKHIVISLVNGARNLVVSGPPQSLYGLNLALRRA     395
NP_012739.1         PMLSISNLTQEQVQDYVNKTNSHLPAGKQVEISLVNGAKNLVVSGPPQSLYGLNLTLRKA     396
XP_451653.1         PMLSISNLTQDQVQTYIDQTNAHLPESKHIVVSLVNGARNLVVSGPPQSLYGLNLTLRKA     396
                    ***:: :*    :: .:: ** :**  ::: ::*:*. :.:**:**  **:**** **:

NP_594370.1         KA-DGQNQSRIPHSKRKLRFINRFLSISVPFHSPYLAPVRSLLEKDLQG----LQFSALK     447
XP_003719712.1      KAPTGLDQNRIPYTQRKVRFVNRFLPITAPFHSKYLAEATSMIDNDLQY--THFEVKDLK     466
XP_962465.1         KAATGLDQTRIPHTERKVRFVNRFLPITAPFHSKYLAKASDLIAEDLKD--VQIDSRTLG     466
NP_984945.2         KAPAGLDQSRIPHSERKLKFTNRFLPIESPFHSHLLEPALELIKQDLHDADLEFKQESLA     455
NP_012739.1         KAPSGLDQSRIPFSERKLKFSNRFLPVASPFHSHLLVPASDLINKDLVKNNVSFNAKDIQ     456
XP_451653.1         KAPAGLDQARIPFSERKLKFFNRFLPIVSPFHSHLLEPANALIEHDLAAAGVEFQASDLK     456
                    **  * :* ***.::**::* **** :  ****  *  .  :: .**      :.   :

NP_594370.1         VPVYSTDDAGDLRFEQPSKLLLALAVMITEKVVHWEEACGFPDVTHIIDFGPGGISGVGS     507
XP_003719712.1      IPVFDTHTGKDIREEVKGNIVPTLIRLITRDPVHWEKATVFPGATHVLDFGPGGISGLGI     526
XP_962465.1         MPVYNTFTGKDLREEVEGNIVPSLIRMITGETVHWEKATVFQDATHILDFGPGGISGLGI     526
NP_984945.2         IAVYDTYDGHDLRQH-SGSIVERIASCITKLPVYWETATTFK-STHILDYGPGGASGLGV     513
NP_012739.1         IPVYDTFDGSDLRVL-SGSISERIVDCIIRLPVKWETTTQFK-ATHILDFGPGGASGLGV     514
XP_451653.1         IPVYDTFNGKDLRES-KEPIASRITYCITSLPVNWEESTKFN-ATHILDFGPGGASGLGV     514
                    : *:.*  . *:*      :   :   *    * ** :  *   **::*:**** **:*

NP_594370.1         LTRANKDGQGVRVIVADSFES---LDMGAKFEIFDRDA-KSIEFAPNWVKLYSPKLVKNK     563
XP_003719712.1      LTSRNKDGTGVRVILAGSVAGTV-TEVGYKPELFDRDEEHAVKYAIDWVKEYGPKLIKTA     585
XP_962465.1         LTSRNKEGTGVRVILAGTVNGTV-TDVGYKPELFDRDEEQAVKFAVNWVKEYGPRLVKNS     585
NP_984945.2         LTHRNKDGTGVRVIIAGALDHNIDDEYGFKQELFDVNP-TSLKFASNWLEEFHPKLIKTG     572
NP_012739.1         LTHRNKDGTGVRVIVAGTLDINPDDDYGFKQEIFDVTS-NGLKKNPNWLEEYHPKLIKNK     573
XP_451653.1         LTHRNKDGTGVRVIIAGALDTNPDDEYGFKQELFDVTS-SGLKFSVNWLEQFHPKLVKTK     573
                    **  **:* *****:*.:.      : * * *:**     .::   :*:: : *:*:*.

NP_594370.1         LGRVYVDTRLSRMLGLPPLWVAGMTPTSVPWQFCSAIAKAGFTYELAGGGYFDPKMMREA     623
XP_003719712.1      TGRTYVDTKMSRLLGLPPVMVAGMTPCTVKWDFVAATMNAGYHIELAGGGYFEPRMMTDA     645
XP_962465.1         VGRTYVDTKMSRLLGLPPLLVAGMTPTTVPWDFIAATMNAGYQIELAGGGYYNAKTMTEA     645
NP_984945.2         SGKVYVDTKFSRLLGRPPLLVPGMTPTTVSPDFVAATINSGYHIELAGGGYFSPQGMTEA     632
NP_012739.1         SGKIFVETKFSKLIGRPPLLVPGMTPCTVSPDFVAATTNAGYTIELAGGGYFSAAGMTAA     633
XP_451653.1         AGKIYVDTTFSKLLGRAPLLVPGMTPTTVSPDFVAATINAGYHIELAGGGYFTAEGMTKA     633
                     *: :*:* :*:::*  *: * **** :*  :* :*  ::*:  *******:    *  *

NP_594370.1         IHKLSLNIPPGAGICVNVIYINPRTYAWQIPLIRDMVAEGYPIRGVTIAAGIPSLEVANE     683
XP_003719712.1      LVKIEKAIPAGRGISVNLIYVNPRAMQWQIPLIGRLRSQGVPIEGLTIGAGVPSIEVANE     705
XP_962465.1         ISKIEKAVPAGRGICVNLIYVNPRAMGWQIPLLGRLRAEGYPIEGLTIGAGVPSIEVAQE     705
NP_984945.2         IDSVVAQIKKGSSLGINLIYVNPRMLQWGIPLIKELRSKGYPIQSLTIGAGVPSLEVASE     692
NP_012739.1         IDSVVSQIEKGSTFGINLIYVNPFMLQWGIPLIKELRSKGYPIQFLTIGAGVPSLEVASE     693
XP_451653.1         IEDVISKIKKGYGLGINMLYVNPRMLQWGIPMIKELREKGYPIQALTIGAGVPSIDVATE     693
                    : .:   :  *  : :*::*:**    * **::  :  :* **. :**.**:**::** *
```

```
NP_594370.1         LISTLGVQYLCLKPGSVEAVNAVISIAKANPTFPIVLQWTGGRAGGHHSFEDFHSPILLT    743
XP_003719712.1      YIQTLGLKHISFKPGSTDAIQAVINIANANPTFPVMLQWTGGRGGGHHSFEDFHQPVLLM    765
XP_962465.1         YIETLGLKHISFKPGSGEAIQAVINIAKANPTFPVILQWTGGRGGGHHSFEDFHHPILNL    765
NP_984945.2         YIETLGMTHLGLKPGSVDAISQVITIAKAHPTFPIVVQWTAGRGGGHHSFENFHAPMLQM    752
NP_012739.1         YIETLGLKYLGLKPGSIDAISQVINIAKAHPNFPIALQWTGGRGGGHHSFEDAHTPMLQM    753
XP_451653.1         YIETLGLTHLGLKPGSIDAISQVIAIAKAHPTFPIVMQWTGGRGGGHHSFEDFHAPILQM    753
                     *.***: :: :**** :*:. ** **:*:*.**: :***.**.*******: * *:*

NP_594370.1         YSAIRRCDNIVLIAGSGFGGADDTEPYLTGEWSAAFKLPPMPFDGILFGSRLMVAKEAHT    803
XP_003719712.1      YNRIRKCDNLILIAGSGFGGAEDTYPYITGEWSRKFGYPPMPFDGCLFGSRVMVAKEAHT    825
XP_962465.1         YGRIRRQENIILVAGSGFGGADDTYPYLTGDWSIKYGYPPMPFDGCMFGSRMMVAKEAHT    825
NP_984945.2         YGKLRRHSNIILIAGSGFGSSEETYPYLTGEWSTRFNYPPMPFDGFLFGSRVMVAKEAKT    812
NP_012739.1         YSKIRRHPNIMLIFGSGFGSADDTYPYLTGEWSTKFDYPPMPFDGFLFGSRVMIAKEVKT    813
XP_451653.1         YAKIRRHSNIYLIAGSGFGSAEDTYPYLTGEWSTKFNYPPMPFDGFLFGSRVMIAKETKT    813
                    *  :*:  *: *: *****.:::* **:**:**  :  ******* :****:*:***.:*

NP_594370.1         SLAAKEAIVAAKGVDDSEWEKTYDGPTGGIVTVLSELGEPIHKLATRGIMFWKELDDTIF    863
XP_003719712.1      APAAKQAIVDAPGVDDDEWEKTYNGVAGGITTVLSEMGEPIHKIATRGVKFWSEMDQKIF    885
XP_962465.1         SLAAKQAIVDAPGLDDSDWEKTYKGPAGGVITVRSEMGEPIHKLATRGVRFWAEMDQKIF    885
NP_984945.2         SLAAKKAIAACTGVPDELWEQTYKKPTGGIVTVRSEMGEPIHKIATRGVMLWKELDETIF    872
NP_012739.1         SPDAKKCIAACTGVPDDKWEQTYKKPTGGIVTVRSEMGEPIHKIATRGVMLWKEFDETIF    873
XP_451653.1         SPATKKTIAECSGVPDSQWEQTYKKPTGGIITVRSEMGEPIHKLATRGVMLWKEMDDTIF    873
                    :  :*: *. . *: *. **:**.  :**: ** **:******:****: :* *:*:.**

NP_594370.1         SLPRPKRLPALLAKKQYIIKRLNDDFQKVYFPAHIVEQVSPEKFKFEAVDSVEDMTYAEL    923
XP_003719712.1      SLPKEKRVPELKKMRSYIIEKLNKDFHKVWFGKNKAGQ----------AVDLEDMTYAEV    935
XP_962465.1         SLPKEKRVAALKASRDYIIEKLNKDFQKVWFGQNKEGK----------AVDLEDMTYAEI    935
NP_984945.2         NLPKNKLQPALDAKRDYIIQKLNSDFQKPWFAT-VNGE----------ARELTDMTYKEV    921
NP_012739.1         NLPKNKLVPTLEAKRDYIISRLNADFQKPWFAT-VNGQ----------ARDLATMTYEEV    922
XP_451653.1         SLPKNKLQAALDAKKDYIISKLNADYQKPWFAT-VNGE----------ARDLADMTYEEV    922
                    .**: *    *   :.***.:** *::* :*      :          . .:  *** *:

NP_594370.1         LYRAIDLMYVTKEKRWIDVTLRTFTGKLMRRIEERFTQDVGKTTLIENFEDLNDPYPVAA    983
XP_003719712.1      VRRMVELMYVKHESRWIDPSFMRLTGDFIHRVEERFVQSSGQPSLLQHYTDLTQPFETIE    995
XP_962465.1         VRRLVELLYVKDEARWIDQSYTKLTGDFIHRIEERFTTTPGQPSRLQSYADLKEPYSAVE    995
NP_984945.2         AERMVELMFIKSTGKWIDVTLRNFTGDFLRRVEERFTKDK-SVSVLQSYSDLETPSKVLA    980
NP_012739.1         AKRLVELMFIRSTNSWFDVTWRTFTGDFLRRVEERFTKSK-TLSLIQSYSLLDKPDEAIE    981
XP_451653.1         AKRMVELMYIRSTNSWIDFTLRNFTGEFLRRVEERFTKQK-TVSIIQNFAVLEEPEKALE    981
                      * ::*:::     *:* :   :**.:::*:****.      : :: :  *  *  .

NP_594370.1         RFLDAYPEASTQDLNTQDAQFFYSLCSNPFQKPVPFIPAIDDTFEFYFKKDSLWQSEDLA    1043
XP_003719712.1      KILAHYPQAEDQLINAQDVQHFLQLCMRPIQKPVVFVPALDDNFDFYFKKDSLWQSEDLQ    1055
XP_962465.1         QILSHYPEAEKQLINAQDVQHFLLLCQRRGQKPVTFVPVLDENFEFFFKKDSLWQSEDLA    1055
NP_984945.2         DIFSAYPAAKSQFVNAQDVAYFLECAQRPTQKPVPFIPVLDHRFEFFFKKDSLWQSENLE    1040
NP_012739.1         KVFNAYPAAREQFLNAQDIDHFLSMCQNPMQKPVPFVPVLDRRFEIFFKKDSLWQSEHLE    1041
XP_451653.1         KVFDAYPAAKEQFLNAQDIDYFLTLCQRPTQKPVPFVPVLDSRFEFFFKKDSLWQSEHLE    1041
                     .:  ** *  * :*:**  .*   . .  **** *:*.:*  *:::**********.*
```

```
NP_594370.1         AVVGEDVGRVAILQGPMAAKHSTKVNEPAKELLDGINETHIQHFIKKFYAGDEKKIPIVE    1103
XP_003719712.1      AVMDQDVQRTCILQGPMAVRYSTVVDEPIQSILDGVHEGHIAGLSKDLYQGKTSTLPSIE    1115
XP_962465.1         AVVGKDVGRTCILQGPMAAKHATKVDEPIKDILDGIHNGHIALLTRDMYGGDESKIPTIE    1115
NP_984945.2         AVVDEDVQRTCILHGPVAAQYTKEINEPIQHILDSIHDGHINKLLKDYYSDDISKVPVVE    1100
NP_012739.1         AVVDQDVQRTCILHGPVAAQFTKVIDEPIKSIMDGIHDGHIKKLLHQYYGDDESKIPAVE    1101
XP_451653.1         AVVDQDVQRTCILHGPVAAQFTNQVDQPIKSILDDVHEGHIKMLLKDYYNNDDSKIPVVE    1101
                    **:.:** *..**:**:*.:.:. :::* : ::*.::: **  : :. * .. ..:* :*

NP_594370.1         YFGGVPPVNVSHK--SLESVSVTEEAGSKVYKLPEIGSNSALPSKKLWFELLAGPEYTWF    1161
XP_003719712.1      YFGGKLMEREFPDVDSIDGLSVSEERGVRDIFRISLSPSQTMPSTDHWLSLIAGPTRSWR    1175
XP_962465.1         YFGGKLIESEIPL--DIEGLTVSYD-EHKNTYRLSSSPSVQLPSLDAWLSLLAGHQRNWR    1172
NP_984945.2         YFGGENPREVDYPTV--------KKTPDTIVYNATS-----ATDFRTWFNVLAGSQKSWR    1147
NP_012739.1         YFGGESPVDVQSQVDS------SSVSEDSAVFKATS-----STDEESWFKALAGSEINWR    1150
XP_451653.1         YFGGEEPEKVAFDS---------IESGNKATYQASS-----STDSSKWFKLLAGEEKTWR    1147
                    ****                                       .   *:. :**   .*

NP_594370.1         RAIFTTQRVAKGWKLEHNPVRRIFAPRYGQRAVVKGKD--NDTVVELYETQSG-NYVLAA    1218
XP_003719712.1      HALLMSEVIVQGTKFQSNPMRRIFAPCRGLFVEVRYPKDPASTQIIVREQPRHNHYVDVL    1235
XP_962465.1         FALLQSDVLVQGQKYQTNPMRRIFAPARGLFVEILHPNNPEKTVILVKEQPRPNRYAEVI    1232
NP_984945.2         HAFFSIPRVVQGSRYSENPTFKVFKPCDNMVVTIKHPETPDKTEVILQEPVQG-TLKTTA    1206
NP_012739.1         HASFLCSFITQDKMFVSNPIRKVFKPSQGMVVEISNGNTSSKTVVTLSEPVQG-ELKPTV    1209
XP_451653.1         SAFFSTPRIVQDSLLIENPARKVFKPSDNMVVQIENAKDSAKTVVTLLETVQG-ELKPTA    1206
                     * :    :.:.     **  ::* *  .  . :   .   .* : : *         .

NP_594370.1         RLSY-DGETIVVSMFENRNALKKEVHLDFLFKYEPSAGYSPVSEILDGRNDRIKHFYWAL    1277
XP_003719712.1      VARLSGKNEIIVELIKDTTALGKPVSLPLKFIYKPEAGYAPLHEVMDDRNDRIKEFYWRA    1295
XP_962465.1         EVKLDGKNEIVVNMIKDTTALGKPVPLTLKFIYNPEAGYAPIHEVMDGRNDRIKEFYWRA    1292
NP_984945.2         TVTF-SGDVIQLSMIENRTMDGIPVALPLLYKYNPTDGFAPIVEIMEDRNQRIKEMYWKL    1265
NP_012739.1         ILKLLKENIIQMEMIENRTMDGKPVSLPLLYNFNPDNGFAPISEVMEDRNQRIKEMYWKL    1269
XP_451653.1         ILRLVKDTLIQLDLIENRTIDGSPVSLPLLFEYNTEDGFAPIAEVTKGRNSRIKEMYWKL    1266
                             * :.:::: .     * * : : ::   *::*: *: ..**.***.:**

NP_594370.1         WFGEEPYPENASITDTFTGPEVTVTGNMIEDFCRTVGNHNEAYTKRAIRKRMAPMDFAIV    1337
XP_003719712.1      WFGDEKLELDANVDSAFDGGKDVVTSEAINDFVHAVGNTGEAFVDRPGKVMYAPMDFAIV    1355
XP_962465.1         WFGDEKLDLDSSVHEVFDGGKTTITSEAINEFVHAVGNTGEAFVDRPDKVMYAPMDFAIV    1352
NP_984945.2         WLK-DPFNLDFDPRDPIESEDFTITSKDIAAFTHAIGNNCEDFVTRPGRPLLAPMDFAIV    1324
NP_012739.1         WID-EPFNLDFDPRDVIKGKDFEITAKEVYDFTHAVGNNCEDFVSRPDRTMLAPMDFAIV    1328
XP_451653.1         WLD-EPFNLDFDPRSVIKGGDFTISAKDISEFVHAIGNNCEDFVARSGREMLAPMDFAIV    1325
                    *:  :    : .  . : . .  ::.: :  * :::**  * :. *  :   ********

NP_594370.1         VGWQAITKAIFPKAIDGDLLRLVHLSNSFRMV-GSHSLMEGDKVTTSASIIAILNNDSGK    1396
XP_003719712.1      VGWKAITKPIFPRKIDGDLLNLVHLSNQFRMKPGAEPLKKGDEVSTVSRVNAVVIQESGK    1415
XP_962465.1         VGWKAITKPIFPRTIDGDLLKLVHLSNQFRMMPGAEPLKKGDVVSTTAQINAVINQESGK    1412
NP_984945.2         IGWRALVKAIFPNNVDGDLLNLVHLSNSYRMIPGAKPLQENDVVNVSALIKSVVNQENGK    1384
NP_012739.1         VGWRAIIKAIFPNTVDGDLLKLVHLSNGYKMIPGAKPLQVGDVVSTTAVIESVVNQPTGK    1388
XP_451653.1         VGWRVIVKAIFPNAVDGDLLKLVHMSNGYRMIPGATPLKEGDTISTSAVIKSVVNQPNGK    1385
                    :**:.: * ***. :*****.***:** ::*  *:  *  .* :.. : : ::: : .**
```

```
NP_594370.1         TVTVKGTVYRDGKEVIEVISRFLYRGTFTDFENTFEHTQETPMQLTLATPKDVAVLQSKS    1456
XP_003719712.1      MVEVCGTITRDGEPVMEVTSQFLYRGTYTDFENTFQSKIETPMQIHLATSKDVAVLRSKE    1475
XP_962465.1         MVEVCGTITRDGEKVMEVTSQFLYRGTYTDFENTFQRKVETPMQLHLATSKDVAVLRSKD    1472
NP_984945.2         LVEVVGTIKKSGKPVIEVTTSFLYRGKYSDFENTFQKSTDPVYVIQINSPKDIAVLKSKE    1444
NP_012739.1         IVDVVGTLSRNGKPVMEVTSSFFYRGNYTDFENTFQKTVEPVYQMHIKTSKDIAVLRSKE    1448
XP_451653.1         IVEVIGTLTRDDKPVMEVTSSFFYRGTYADYENTFEKTVEPIYQIEVKSSKDSAVLRSKE    1445
                     * * **: :..: *:** : *:***.::*:****: . :    : : : ** ***:**.

NP_594370.1         WFQLLD---PSQDLSGSILTFRLNSYVRFKDQKVKSSVETKGIVLSELPSKAIIQVASVD    1513
XP_003719712.1      WFTLEDN--VNMDLLGQTLIFRLQSLVRFKNTTVFSSVQTRGEVLVELPTKEIVQVGSVE    1533
XP_962465.1         WFMMDDVENPDIELLGQTLTFRLQTLVRFKNKTVFSHVETQGQVLLELPTKEIIQVATVN    1532
NP_984945.2         WLHFDN---DKIDLLGRTLTFETETEVTFKNRTVFSSVHCEGRVLMELSTKENVQIGVVK    1501
NP_012739.1         WFQLDD---EDFDLLNKTLTFETETEVTFKNANIFSSVKCFGPIKVELPTKETVEIGIVD    1505
XP_451653.1         WFQLDD---EEIDLLGKTLSFQTETEVTFKNASVFSSVKCQGKILMELPTKETVEIGSVD    1502
                    *: : :    . :* .  * *. :: * **: .: * *.  * :  ** :*  :::. *.

NP_594370.1         FQSVDCHGNPVIEFLKRNGKPIEQPVEFENGGYSVIQVMDEGYSPVFVTPPTNSPYAEVS    1573
XP_003719712.1      YEAGTSHGNPVIDFLERNGSSIEQPINFENPIPLSGKT-----PLQLRAPASNETYARVS    1588
XP_962465.1         YYAGESHGNPVIDYLQRHGSSIEQPINFENPIPLSGKT-----PLQLKAPSSNETYARVS    1587
NP_984945.2         YDAGESHGNPVIDYLSRNGSTLEHKVNLENTIPIAT--------IEAQSPGTNETYARVS    1553
NP_012739.1         YEAGASHGNPVVDFLKRNGSTLEQKVNLENPIPIAV--------LDSYTPSTNEPYARVS    1557
XP_451653.1         YEAGESHGNPVIDYLSRNGSTLEQKVNLENAIPIAV--------RDTQSPSTNEPYARVS    1554
                    : :  .*****:::*.*:*. :*: :::**                  :* :*. **.**

NP_594370.1         GDYNPIHVSPTFAAFVELPGTHGITHGMYTSAAARRFVETYAAQNVPERVKHYEVTFVNM    1633
XP_003719712.1      GDYNPIHVSRVFANYADLPGT--ITHGMYSSAAVRSLVETWAAENDNSRVRSFHASLVGM    1646
XP_962465.1         GDYNPIHVSRVFSSYANLPGT--ITHGMYSSAAVRSLVETWAAENKVGRVRSFHASLTGM    1645
NP_984945.2         GDLNPIHVSRHFANYADLPGT--ITHGMYTSGAVRALVETWAADSVSSRVRAYSCQFVGM    1611
NP_012739.1         GDLNPIHVSRHFASYANLPGT--ITHGMFSSASVRALIENWAADSVSSRVRGYTCQFVDM    1615
XP_451653.1         GDLNPIHVSRHFAAYANLPGT--ITHGMYSSATVRALVETWAADNVSSRVRAYNCQFVGM    1612
                    ** ******  *: :.:****  *****::*.:.* ::*.:**:.   **: :   :..*

NP_594370.1         VLPNTELITKLSHTGMINGRKIIKVEVLNQETSEPVLVGTAEVEQPVSAYVFTGQGSQEQ    1693
XP_003719712.1      VLPNDDLLVKLQHVGMVAGRKIIKVEVINKETEDKVLLGEAEVEQPVTAYVFTGQGSQEQ    1706
XP_962465.1         VLPNDDINVKLQHVGMVAGRKIIKVEASNKETEEKVLLGEAEIEQPITAYIFTGQGSQEQ    1705
NP_984945.2         VLPNTLLTTRIEHVGMINGRKLIKFETRND-KDEPVLAGEAEVQQPVSTFVFTGQGSQEQ    1670
NP_012739.1         VLPNTALKTSIQHVGMINGRKLIKFETRNE-DDVVVLTGEAEIEQPVTTFVFTGQGSQEQ    1674
XP_451653.1         VLPNTPLQTTIQHVGMINGRKLIQFETKND-ADETVMTGEAEVEQPVSTFVFTGQGSQEQ    1671
                    ****  : . :.*.**: ***:*:.*. *.  .  *: * **::**:::::*********

NP_594370.1         GMGMDLYASSPVARKIWDSADKHFLTNYGFSIIDIVKHNPHSITIHFGGSKGKKIRDNYM    1753
XP_003719712.1      GMGMELYESSPVAKEVWDRADKYLMDTYGFAITNIVKNNPKELTIHFGGPRGKAIRANYM    1766
XP_962465.1         GMGMDLYNSSPVAKAVWDRADKYLMDTYGFAITNIVRNNPKELTIHFGGPRGKAIRQNYM    1765
NP_984945.2         GMGMDLYDKSPVARQVWDRADNHFKQTYGFSILEIVRTNPKELTIYFGGEKGRKIKENYT    1730
NP_012739.1         GMGMDLYKTSKAAQDVWNRADNHFKDTYGFSILDIVINNPVNLTIHFGGEKGKRIRENYS    1734
XP_451653.1         GMGMDLYAKSDVAKQVWDRADQHFKSTYGFSILDIVKNNPTELTIYFGGEKGRKIKENYT    1731
                    ****:** .* .*: :*: **:::  .***:* :**  ** .:**:*** :*: *: **
```

```
NP_594370.1         AMAYEKLMEDGTSKVVPVFETITKDSTSFSFTHPSGLLSATQFTQPALTLMEKSAFEDMR        1813
XP_003719712.1      AMTFETVAADGTIKSERIFKDINERTTSYTYRSPTGLLSATQFTQPALTLMEKASFEDMK        1826
XP_962465.1         SMTFETVAADGSIKSERMFKEIDEKTTSYTYRSPNGLLSATQFTQPALTLMEKASFEDMK        1825
NP_984945.2         SMIFETI-VDGEIASERIFKSITESSTSYTFKSDTGLLSATQFTQPALTLMEKASFEDLK        1789
NP_012739.1         AMIFETI-VDGKLKTEKIFKEINEHSTSYTFRSEKGLLSATQFTQPALTLMEKAAFEDLK        1793
XP_451653.1         QMIFETI-VDGEVKSERIFKDITEETNSFTFRSPTGLLSATQFTQPALTLMEKASFEDLK        1790
                     * :*.:  **      :*: * : :.*:::   .******************::***::

NP_594370.1         SKGLVQNDCAFAGHSLGEYSALSAMGDVLSIEALVDLVFLRGLTMQNAVHRDELGRSDYG        1873
XP_003719712.1      AKGLVPRDSTFAGHSLGEYSALAALADVMPIESLVSVVFYRGLTMQVAVERDASGRSNYS        1886
XP_962465.1         AKGLVPRDSTFAGHSLGEYSALAALADVMPIESLVSVVFYRGLTMQVAVERDETGRSNYG        1885
NP_984945.2         SKGLIPAEATFAGHSLGEYAALASLADVMSIESLVEVVFYRGMTMQVAVPRDSEGRSNYG        1849
NP_012739.1         SKGLIPADATFAGHSLGEYAALASLADVMSIESLVEVVFYRGMTMQVAVPRDELGRSNYG        1853
XP_451653.1         SKGLIPAEATFAGHSLGEYAALASLADVMSIESLVEVVFYRGMTMQVAVPRDSEGRSNYG        1850
                    :***:  :.:*********:**:::.**: **:**.:** **:*** ** **  ***:*.

NP_594370.1         MVAANPSRVSASFTDAALRFIVDHIGQQTNLLLEIVNYNVENQQYVVSGNLLSLSTLGHV        1933
XP_003719712.1      MCAVNPSRISKTFNEEALRFVVGNIAEGTGWLLEIVNYNIANMQYVCAGDLRALDTLTNV        1946
XP_962465.1         MCAVNPSRINKTFTEEALRFVVGSIAETTGWLLEIVNFNVENMQYVCAGDLRALDTLGGV        1945
NP_984945.2         MIAVNPSRVSTGFTQEALQFVVERVGKITEWLVEIVNYNVEDQQYVAAGDLRALDTLTNV        1909
NP_012739.1         MIAINPGRVAASFSQEALQYVVERVGKRTGWLVEIVNYNVENQQYVAAGDLRALDTVTNV        1913
XP_451653.1         MIAVNPSRVSPTFSQEALEFVVKIVDKKTGWLLEIVNYNVENQQYVAAGDLRALDTLTNV        1910
                    * * **.*:   *.: **.::*  : : *  *:****:*: : *** :*:* :*.*:  *

NP_594370.1         LNFLKVQKIDFEKLKETLTIEQLKEQLTDIVEACHAKTLEQQKKTGRIELERGYATIPLK        1993
XP_003719712.1      LNYLKAQKIDIEAMKAAMTVEDVKQHLQEIIKASAEKTE---AKPKPLELERGFATIPLR        2003
XP_962465.1         CNAIKMMKIDIEKMRKEYSAETVKEQLVEIIKSCANDTQ---AKPTPLVLQRGFATIPLP        2002
NP_984945.2         LNFVKLQKIDLVKLQATMPLEQVEEHLDEIITEISKKSL---SKPQPIELERGFACIPLR        1966
NP_012739.1         LNFIKLQKIDIIELQKSLSLEEVEGHLFEIIDEASKKSA---VKPRPLKLERGFACIPLV        1970
XP_451653.1         LNFVKLQKIDIAKLQETLSLEEVESHLNEIIEEISKKST---AKPQPIELERGFACIPLR        1967
                     * :*  ***:  ::     * :: :* :*:     .:     *   : *:**:* ***

NP_594370.1         -IDVPFHSSFLRGGVRMFREYLVKKIFPHQINVAKLRGKYIPNLTAKPFEISKEYFQNVY        2052
XP_003719712.1      GIDVPFHSTFLRSGVKPFRSFLLKKINKTTIDPSKLLGKYIPNVTAKPFALTKEYFEDVY        2063
XP_962465.1         GIDVPFHSTFLRSGVKPFRSFLLKKINKTTIDPSKLIGKYIPNVTAKPFAITKEYFEDVY        2062
NP_984945.2         GISVPFHSSYLRNGVKPFKNFLKKNIIKENVKVDRLIGKYIPNLTAKPFAITKEYFEDVY        2026
NP_012739.1         GISVPFHSTYLMNGVKPFKSFLKKNIIKENVKVARLAGKYIPNLTAKPFQVTKEYFQDVY        2030
XP_451653.1         GISVPFHSSYLRNGVKPFKNFLMKNVTKENVKADRLIGKYIPNLTAKPFEITKEYFQDVY        2027
                     *.*****.:* .**: *:.:* *::    :.  :* ******:***** ::****::**

NP_594370.1         DLTGSQRIKKILQNWDEYESS------------------------------------   2073
XP_003719712.1      RLTNSPRIASILANWEQYQNENAVPSTIGLGANGAGDANGETTENGVEEANGANGVH   2120
XP_962465.1         RLTNSPKIQQILANWDKYQEEGVSGIP---------DAE------------------   2092
NP_984945.2         KLTGSEKIKAVLDNWESYQN-------------------------------------   2046
NP_012739.1         DLTGSEPIKEIIDNWEKYEQS------------------------------------   2051
XP_451653.1         ELTGSEKIKDILDNWEKITN-------------------------------------   2047
                     **.*  *  :: **:.  .
```

Figure S2. Multiple sequence alignment of 5 homologous of Ascomycota (FAS1 *Saccharomyces cerevisiae*, uncharacterized protein *Kluyveromyces lactis*, AER085Cp *Eremothecium gossypii*, FAS1 *Schizosaccharomyces pombe*, and FAS1 *Neurospora crassa*, FAS1 *Magnaporthe oryzae*).

Table S2. Table to show ascomycetes fungus strain, phylum, substrate, host and country which are closely related to *Magnaporthe oryzae* S6 Malaysian strain. The colour dot indicated for each species can be matched with phylogenetic (Figure 3) to recognized the position of particular ascomycetes in the phylogenetic tree using the information in NCBI and fungal related journals [29-32].

Gene	Colour in Phylogenetic	Species	Strain	Phylum	Subtrate	Type of host	Country	Accession No	Percentage Identity (%)
**Fatty acid synthase beta subunit dehydratase**	●	*Magnaporthe oryzae* S6	S6	Ascomycetes	–	Paddy	Malaysia	–	100.00
**Fatty acid synthase beta subunit dehydratase**	●	*Pyricularia oryzae 70-15 [37,47,49]*	70-15	Ascomycetes	–	Paddy, Wheat,Millet	Asia, South America	XP_003719712.1	100.00
**Fatty acid synthase beta subunit dehydratase**	●	*Pyricularia oryzae* Y34	Y34	Ascomycetes	*Oryza sativa L. ssp. indica*	Paddy	Yunnan, China	ELQ41749.1	99.88
**Hypothetical protein PspLS_06761**	●	*Pyricularia sp.CBS 133598*	NI919	Ascomycetes	*Leersia oryzoides*	Rice cutgrass	Chiba, Japan	TLD23790.1	98.73
**Uncharacterized PgNI_06784**	●	*Pyricularia grisea* [48]	NI907	Ascomycetes	*Digitaria sanguinalis, Eleusine coracana*	Crabgrass, Finger Millet	Tochigi, Japan, Eastern Africa	XP_030980725.1	98.50
**Hypothetical protein PpBr36_04604**	●	*Pyricularia pennisetigena*	Br36	*Ascomycetes*	*Cenchrus echinatus*		Parana, Brazil	XP_029750409.1	98.27
**Fatty acid synthase beta subunit dehydratase**	●	*Magnaporthiopsis poae* ATCC 64411[36]	ATCC 64411	*Ascomycetes*	*Poa pratensis*	Kentucky Bluegrass	North America	KLU82571.1	86.46
**Fatty acid synthase beta subunit dehydratase**	●	*Gaeumannomyces tritici* R3-111a-1	R3-111a-1	*Ascomycetes*	–	–	–	XP_009219081.1	86.17
**Putative fatty acid synthase beta subunit dehydratase**	●	*Phaeocremonium minimum* UCRPA7	UCRPA7	*Ascomycetes*	*Vitis vinifera cv. Thompson*	Wood canker	Fresno County, California	XP_007917534.1	80.07
**Malonytransferase-like protein**	●	*Coniella lustricola*	B22-T-1	*Ascomycetes*	–	–	–	PSS00700.1	78.52
**Putative fatty acid synthase beta subunit dehydratase**	●	*Diaporthe ampelina*	DA912	*Ascomycetes*	*Vitis vinifera*	Wood from a vineyard	Solano County, California	KKY38508.1	79.02

**Chain elongation-2**		*Diaporthe helianthi*	7/96	*Ascomycetes*	–	–	France	POS73465.1	78.50
**Hypothetical protein VPNG_02967**		*Cytospora leucostoma*	VPNG_02967	*Ascomycetes*	Peach	Peach orchard	Shaanxi, China	VPNG_02967	80.58
**Fatty acid synthase beta**		*Valsa mali var. pyri*	SXYL134	*Ascomycetes*	Apple	Apple orchard	Shaanxi, China	KUI55013.1	80.51
**Fatty acid synthase subunit beta**		Valsa mali	03-8	*Ascomycetes*	Apple	Apple orchard	Shaanxi, China	KUI68140.1	80.38
**Hypothetical protein VMCG_05230**		*Valsa malicola*	03-1	*Ascomycetes*	Apple	Apple orchard	Shaanxi, China	ROW5693.1	80.54
**Hypothetical protein VSDG_02422**		*Valsa sordida*	YSFL	*Ascomycetes*	Poplar	Poplar tree	Shaanxi, China	ROW02228.1	80.47
**Beta subunit of fatty acid synthetase**		*Coniochaeta pulveracea*	CAB683	*Ascomycetes*	*Acacia*	Decaying wood	Northern Cape, South Africa	RKU48976.1	78.94
**Beta subunit of fatty acid synthase**		*Coniochaeta lignaria* NRRL 30616	NRRL 30616	*Ascomycetes*	–	–	–	OIW28344.1	80.57
**Fatty acid synthase beta subunit dehydratase**		*Neurospora crassa* OR74A	OR74A	*Ascomycetes*	–	–	–	XP_011394235.1	99.86
**Malonyltransferase-like protein**		*Chaetomium thermophilum var. thermophilum*	DSM 1495	*Ascomycetes*	–	–	–	XP_006694197.1	77.86
**Fatty acid synthase subunit beta**		*Madurella mycetomatis*	mm55	*Ascomycetes*	*Homo sapien*	Human	Khartoum, Sudan	KXX77519.1	79.34
**Uncharacterized protein MYCTH_2310228**		*Thermothelomyces thermophilus* ATCC 42464	ATCC 42464	*Ascomycetes*	–	–	–	XP_003665953.1	78.80
**Conserved hypothetical protein**		*Chaetomium globosum* CBS 148.51	CBS 148.51	*Ascomycetes*	–	–	–	XP_001223166.1	79.09
**Hypothetical protein M434DRAFT_37678**		*Hypoxylon sp.* CO27-5	CO27-5	*Ascomycetes*	–	–	–	OTA81100.1	78.48
**Fatty acid synthase-like protein subunit beta**		*Hypoxylon sp.* EC38	EC38	*Ascomycetes*	–	–	–	OTA69323.1	78.52

**Fatty acid synthase beta subunit dehydratase**	🟡	*Grosmania clavigera* kw1407	kw 1407	*Ascomycete*	–	–	–	XP_014 17809.1	78.98
**Fatty acid synthase beta subunit dehydratase**	🟡	*Sporothrix insectorum* RCEF 264	RCEF 264	*Ascomycetes*	*Lepidopteran*	Larva	Qinghai, China	OAA56 953.1	79.36
**Fatty acid synthase beta subunit dehydratase**	🟡	*Ophiostoma piceae UAMH* 11346	UAMH 11346	*Ascomycetes*	*Pinus contorta*	Lodgepole pine	British Columbia, Canada	EPE035 82.1	78.51
**Fatty acid synthase subunit beta**	🟡	*Sporothrix brasiliensis* 5110	5110	*Ascomycetes*	*Felis catus*	Cat	–	KIH888 60.1	78.49
**Fatty acid synthase subunit beta**	🟡	*Sporothrix schenckii* 1099-18	1099-18	*Ascomycetes*	*Homo sapien*	Human	New York, USA	XP_016 582851.1	78.74
**Fatty acid synthase subunit beta**	🟢	*Colletotrichum sidae* CBS 518.97	CBS 518.97	*Ascomycetes*	*Sida spinosa*	Flowering plant	Arkansas, USA	TEA19 476.1	78.48
**Fatty acid synthase subunit beta**	🟢	*Colletotrichum orbiculare* MAFF 240422	104-T	*Ascomycetes*	*Cucumis sativus*	Cucumber plant leaves	Kyoto, Japan	TDZ22 651.1	78.34
**Fatty acid synthase subunit beta**	🟢	*Colletotrichum trifolii*	543-2	*Ascomycetes*	*Medicago sativa*	Alfalfa or lucerne	Chiba, Japan	TDZ61 178.1	78.48
**Fatty acid synthase subunit beta**	🟢	*Colletotrichum spinosum*	CBS 515.97	*Ascomycetes*	*Xanthium spinosum*	Flowering plant	New South Wales, Australia	TDZ30 993.1	78.43
**Fatty acid synthase subunit beta 1**	🟢	*Colletotrichum chlorophyti*	NTL11	*Ascomycetes*	*Solanum lycopersicum*	Tomato plant	Honshu, Japan	OLN97 389.1	79.16
**Acyl transferase**	🟢	*Colletotrichum salicis*	CBS 607.94	*Ascomycetes*	*Salix sp.*	Willows leaf	Netherlands	KXH27 754.1	78.38
**Acyl transferase**	🟢	*Colletotrichum fioriniae* PJ7	PJ7	*Ascomycetes*	*Fragaria x ananassa*	Strawberry	New Zealand	EXF77 797.1	78.38
**Acyl transferase**	🟢	*Colletotrichum orchidophilum*	IMI 309357	*Ascomycetes*	*Phalaenopsis sp.*	Moth orchid	United Kingdom	XP_022 468818.1	78.24
**Acyl transferase**	🟢	*Colletotrichum higginsianum* IMI 349063	IMI 349063	*Ascomycetes*	***Brassica rapa subsp. chinensis***	–	**Trinidad and Tobago, Trinidad**	XP_018 151290.1	77.97

**Fatty acid synthase subunit beta**	●	*Colletotrichum tanaceti*	BRIP57314	*Ascomycetes*	***Tanacetum cinerariifolium***	**Flowering plant leaf**	**Parkville, Australia**	TKW54859.1	77.93
**Acyl transferase domain-containing protein**	●	*Colletotrichum graminicola* M1.001	M1.001	*Ascomycetes*	–	–	–	XP_008095042.1	78.67
**Putative acyl transferase domain-containing protein**	●	*Colletotrichum sublineola*	TX430BB	*Ascomycetes*	*Sorghum bicolor*	Great millet	Texas, USA	KDN63499.1	78.35
**Fatty acid synthase beta subunit dehydratase (acyl transferase)**	●	*Colletotrichum tofieldiae*	0861	*Ascomycetes*	*Arabidopsis thaliana*	Flowering weed	Las Rozas, Spain	KZL72973.1	78.14
**Fatty acid synthase beta subunit dehydratase, partial**	●	*Colletotrichum incanum*	MAFF 238704	*Ascomycetes*	*Raphanus sativus L.*	Radish	Miyazaki Prefecture, Japan	KZL80018	78.18
**Fatty acid synthase beta subunit dehydratase**	●	*Colletotrichum incanum*	MAFF 238712	*Ascomycetes*	*Raphanus sativus var. longipinnatus*	Mild-flavored winter radish	Kagoshima, Japan	OHW94742.1	78.18
**Probable fatty-acyl-CoA synthase**	●	*Rhynchosporium commune*	UK7	*Ascomycetes*	–	–	–	CZT04859.1	78.32
**Probable fatty-acyl-CoA synthase, beta subunit**	●	*Rhynchosporium secalis*	02CH4-6a.1	*Ascomycetes*	–	–	–	CZT51067.1	78.22
	●	*Rhynchosporium agropyri*		*Ascomycetes*	–	–	–		78.27
**Fatty acid synthase-like protein subunit beta**	●	*Cadophora sp.* DSE 1049	DSE 1049	*Ascomycetes*	–	–	–	PVH81929.1	78.84
**Fatty acid synthase-like protein subunit beta**	●	*Phialocephala scopiformis*	CBS 120377	*Ascomycetes*	–	–	–	XP_018068695.1	78.91
**Fatty acid synthase-like**	●	*Hyaloscypha variabilis* F	F	*Ascomycetes*	–	–	–	PMD46531.1	78.02

**protein subunit beta**									
**Fatty acid synthase-like protein subunit beta**	●	*Coleophoma crateriformis*	BP5796	*Ascomycetes*	–	Leaf	Toyama-ken, Japan	RDW64070.1	78.75
**Fatty acid synthase-like protein subunit beta**	●	*Coleophoma cylindrospora*	BP6252	*Ascomycetes*	–	Leaf	Nagasaki Prefecture, Japan	RDW77183.1	78.56

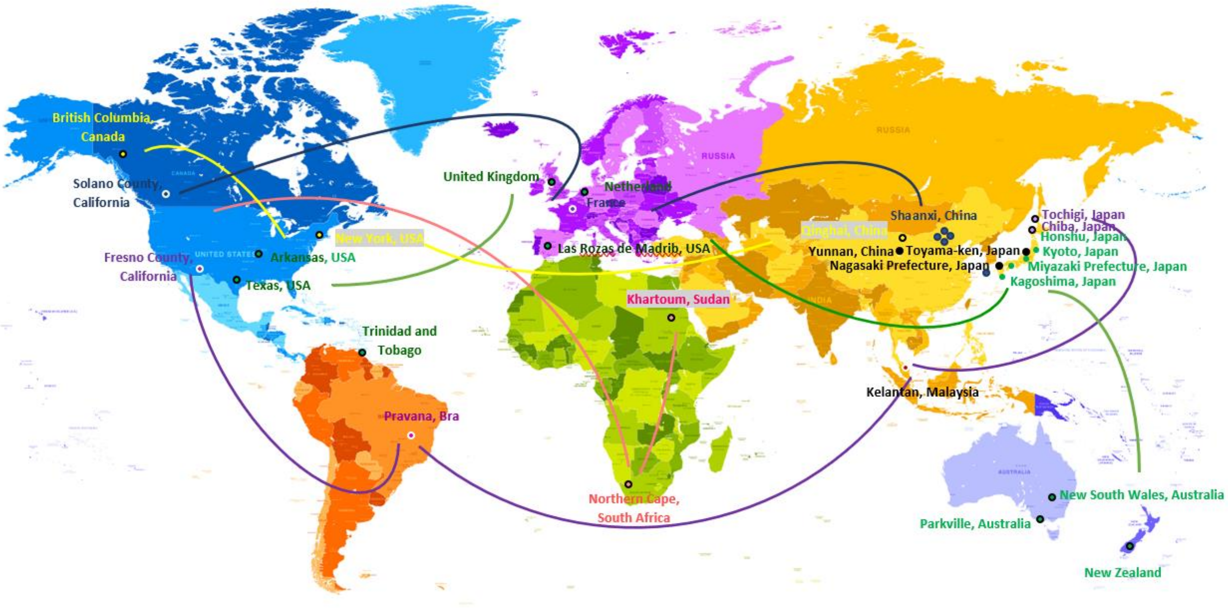

Figure S3. Geographical distribution of ascomycetes, which are closely related to *Magnaporthe oryzae* S6. The color dots in the phylogenetic tree (Figure 3) matches the color codes of their geographical location.

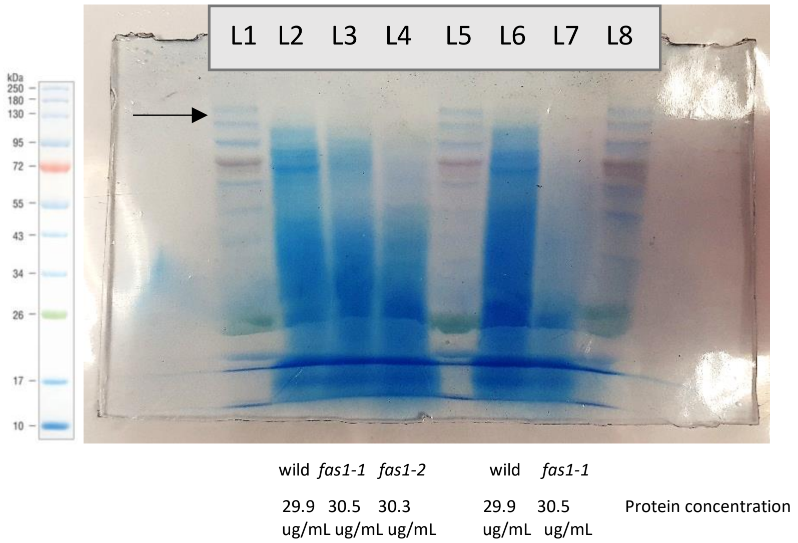

Figure S4. SDS-PAGE. A Lane 1,5,8: Color prestained protein standard, broad range; Lane 2 & 6: Protein extracted from *M. oryzae* S6 wildtype concentration 29.9 ug/mL ; Lane 3 & 7: Protein extracted from *fas1-1* concentration 30.5 ug/mL; Lane 4: Protein extracted from *fas1*-concentration 30.3 ug/mL.

Table S3. (A) Diameter measurement of wildtype, *Δfas1-1* and *Δfas1-2* mutants of three biological replicates of each different carbon sources, 10 days culture under on complete media agar plates at temperature 26 ° C. (B) Average diameter and standard deviation of the replicates in Table S3A. (C) p-value of the diameter measurement of wildtype, *Δfas1-1* and *Δfas1-2* mutants of three biological replicates was determined by calculating the significant value using two way anova with replication.

A

			**Wild Type**		
**Media**	1st	2nd	3rd	Mean	Standard Deviation
**CM**	7.90	8.30	7.90	8.03	0.23
**MM + G**	7.10	7.00	7.00	7.03	0.06
**MM + OO**	6.30	6.25	5.95	6.17	0.19
**MM + OA**	5.30	5.20	4.80	5.10	0.26
**PDA**	7.60	7.70	7.70	7.67	0.06
**MM + SA**	0.40	0.40	0.40	0.40	0.00
			fas1-1		
**Media**	1st	2nd	3rd	Mean	Standard Deviation
**CM**	7.60	7.55	7.80	7.65	0.13
**MM + G**	6.40	6.30	6.80	6.50	0.26
**MM + OO**	5.70	5.60	5.30	5.53	0.21
**MM + OA**	2.75	2.70	2.75	2.73	0.03
**PDA**	7.40	7.40	7.70	7.50	0.17
**MM + SA**	0.40	0.40	0.40	0.40	0.00
			fas1-2		
**Media**	1st	2nd	3rd	Mean	Standard Deviation
**CM**	7.80	7.60	7.70	7.70	0.10

**MM + G**	6.90	6.90	6.90	6.90	0.00
**MM + OO**	6.20	6.10	6.20	6.17	0.06
**MM + OA**	4.40	4.20	4.60	4.40	0.20
**PDA**	7.50	7.60	7.40	7.50	0.10
**MM + SA**	0.40	0.40	0.40	0.40	0.00

B

Sample	Wild	*fas1-1*	*fas1-2*
**CM**	8.03 ± 0.23	7.65 ± 0.13	7.70 ± 0.10
**MM + G**	7.03 ± 0.06	6.50 ± 0.26	6.90 ± 0.00
**MM + OO**	6.17 ± 0.19	5.53 ± 0.21	6.17 ± 0.06
**MM + OA**	5.10 ± 0.26	2.73 ± 0.03	4.40 ± 0.20
**PDA**	7.60 ± 0.06	7.50 ± 0.17	7.50 ± 0.10
**MM + SA**	0.40 ± 0.00	0.40 ± 0.00	0.40 ± 0.00

C

Media	Significant	P value	Mean of wild	Mean of fas1-1	Difference	SE of difference	t ratio	df	Adjusted P value
CM	No	0.0671	8.033	7.650	0.3833	0.1537	2.495	4.000	0.1298
MM+G	Yes	0.0270	7.033	6.500	0.5333	0.1563	3.411	4.000	0.0788
MM+OO	Yes	0.0176	6.167	5.533	0.6333	0.1624	3.899	4.000	0.0684
MM+OA	Yes	0.0001	5.100	2.733	2.367	0.1537	15.40	4.000	0.0005
PDA	No	0.1890	7.667	7.500	0.1667	0.1054	1.581	4.000	0.1890
MM+AA			0.4000	0.4000	0.000	0.000			
Media	Significant	P value	Mean of wild	Mean of fas1-2	Difference	SE of difference	t ratio	df	Adjusted P value
CM	No	0.083474	8.033	7.700	0.3333	0.1453	2.294	4.000	0.187224
MM+G	Yes	0.016130	7.033	6.900	0.1333	0.03333	4.000	4.000	0.078090
MM+OO	No	>0.99999	6.167	6.167	0.000	0.1143	0.000	4.000	>0.999999
MM+OA	Yes	0.021664	5.100	4.400	0.7000	0.1915	3.656	4.000	0.083879
PDA	No	0.066767	7.667	7.500	0.1667	0.06667	2.500	4.000	0.187224
MM+AA	No		0.4000	0.4000	0.000	0.000		4.000	

Table S4. (A) Mycelial dry weight wildtype, *Δfas1-1* and *Δfas1-2* mutants of three biological replicates in each different carbon sources that were grown for 10 days under (12 hours day light, 12 hours dark) on complete media agar plates at temperature 26 º C. (B) Average mycelial dry weight and standard deviation of the replicates in Table S4A. (C) P-value of the mycelium dry weight of wildtype, *Δfas1-1* and *Δfas1-2* mutants of three biological replicates was determined by calculating the significant value using two way anova with replication.

A

**Wild Type**					
**Media**	1st	2nd	3rd	Mean	Standard Deviation
**CM**	9.89	8.65	9.27	9.27	0.62
**MM + G**	8.67	0.86	1.00	8.84	0.17
**MM + OO**	5.20	4.80	5.00	5.00	0.20
**MM + OA**	4.70	0.76	0.86	4.10	0.60
**PDA**	7.60	2.01	1.50	7.48	0.13
fas1-1					
**Media**	1st	2nd	3rd	Mean	Standard Deviation
**CM**	1.28	1.15	1.22	1.22	0.07
**MM + G**	0.86	0.80	0.80	0.82	0.03
**MM + OO**	1.21	1.80	1.51	1.51	0.30
**MM + OA**	0.76	0.50	0.63	0.63	0.13
**PDA**	2.01	1.84	1.93	1.93	0.08
fas1-2					
**Media**	1st	2nd	3rd	Mean	Standard Deviation
**CM**	1.90	1.85	1.88	1.88	0.02
**MM + G**	1.00	0.70	0.70	0.80	0.17
**MM + OO**	3.01	2.20	2.61	2.61	0.41
**MM + OA**	0.86	0.81	0.84	0.84	0.03
**PDA**	1.50	0.97	1.24	1.24	0.27

B

**Sample**	**CM**	**Glucose**	**Olive Oil**	**Oleic Acid**	**PDA**
**Wild**	9.27 ± 0.62	8.84 ± 0.17	5.00 ± 0.20	4.10 ± 0.60	7.48 ± 0.13
fas1-1	1.22 ± 0.07	0.82 ± 0.03	1.51 ± 0.30	0.63 ± 0.13	1.93 ± 0.08
Fas1-2	1.88 ± 0.02	0.70 ± 0.17	2.61 ± 0.41	0.84 ± 0.03	1.24 ± 0.27

C

Media	Significant	P value	Mean of wild	Mean of fas1-1	Difference	SE of difference	t ratio	df	Adjusted P value
CM	Yes	<0.0001	9.270	1.217	8.053	0.3599	22.38	4.000	0.0001
MM+G	Yes	<0.0001	8.840	0.8200	8.020	0.1002	80.07	4.000	<0.0001
MM+OO	Yes	<0.0001	5.000	1.507	3.493	0.2058	16.98	4.000	0.0004
MM+OA	Yes	0.0006	4.100	0.6300	3.470	0.3544	9.790	4.000	0.0031
PDA	Yes	<0.0001	7.477	1.927	5.550	0.08731	63.57	4.000	<0.0001

Media	Significant	P value	Mean of wild	Mean of fas1-2	Difference	SE of difference	t ratio	df	Adjusted P value
CM	Yes	<0.0001	9.270	1.877	7.393	0.3583	20.64	4.000	0.0002
MM+G	Yes	<0.0001	8.840	0.8000	8.040	0.1401	57.38	4.000	<0.0001
MM+OO	Yes	0.0008	5.000	2.607	2.393	0.2608	9.177	4.000	0.0039
MM+OA	Yes	0.0007	4.100	0.8367	3.263	0.3467	9.412	4.000	0.0036
PDA	Yes	<0.0001	7.477	1.237	6.240	0.1692	36.88	4.000	<0.0001

Table S5. Number of conidia harvested from a 9 cm oatmeal agar at day 10 after incubation at 26°C.

**Sample**	**Oatmeal (cell/mL)**	**PDA (cell/mL)**
**Wild**	$2.0 \pm 0.5 \times 10^4$	5.0 ± 2.1 x 10^4
fas1-1	0.25 ± 1.70 x 10^4	1.0 ± 1.50 x 10^4
fas1-2	0.5 ± 2.4 x 10^4	1.0 ± 2.11 x 10^4

Table S6. Size measurement of conidia length and width of 30 conidia in micrometre, wildtype, *Δfas1-1* and *Δfas1-2* mutants that were grown for 10 days under (12 hours day light, 12 hours dark) on complete media agar plates at temperature 26 ° C.

Wild Type					
	No	Measured Conidia Length (cm)	Actual Conidia length (µm)	Measured Conidia (cm)	Conidia width (µm)
	1	1.40	26.9	0.50	9.6
	2	1.60	30.8	0.45	8.7
	3	1.35	26.0	0.45	8.7
	4	1.50	28.8	0.40	7.7
	5	1.90	36.5	0.50	9.6
	6	1.20	23.1	0.50	9.6
	7	1.35	26.0	0.45	8.7
	8	1.60	30.8	0.50	9.6
	9	1.40	26.9	0.45	8.7
	10	1.50	28.8	0.35	6.7
	11	1.80	34.6	0.40	7.7
	12	1.60	30.8	0.40	7.7
	13	1.60	30.8	0.45	8.7
	14	1.80	34.6	0.45	8.7
	15	1.50	28.8	0.40	7.7
	16	1.40	26.9	0.45	8.7
	17	1.85	35.6	0.40	7.7
	18	1.65	31.7	0.40	7.7
	19	1.60	30.8	0.35	6.7
	20	2.00	38.5	0.40	7.7
	21	1.60	30.8	0.35	6.7
	22	1.90	36.5	0.40	7.7
	23	1.65	31.7	0.40	7.7
	24	1.80	34.6	0.40	7.7
	25	2.10	40.4	0.35	6.7
	26	2.00	38.5	0.35	6.7
	27	1.70	32.7	0.45	8.7
	28	2.10	40.4	0.40	7.7
	29	2.10	40.4	0.40	7.7
	30	1.80	34.6	0.45	8.7
		Average	32.3	Average	8.1
		Standard Deviation	4.7	Standard Deviation	0.9

fas1-1				
No	Measured Conidia Length (cm)	Actual Conidia length (µm)	Measured Conidia	Conidia width (µm)
1	1.60	30.8	0.50	9.6
2	1.80	34.6	0.40	7.7
3	1.80	34.6	0.50	9.6
4	1.30	25.0	0.50	9.6
5	1.60	30.8	0.40	7.7
6	1.85	35.6	0.55	10.6
7	1.40	26.9	0.50	9.6
8	1.40	26.9	0.50	9.6
9	1.70	32.7	0.40	7.7
10	1.60	30.8	0.40	7.7
11	1.60	30.8	0.40	7.7
12	1.50	28.8	0.40	7.7
13	1.40	26.9	0.40	7.7
14	1.30	25.0	0.40	7.7
15	1.70	32.7	0.40	7.7
16	1.80	34.6	0.35	6.7
17	1.90	36.5	0.35	6.7
18	1.70	32.7	0.40	7.7
19	1.80	34.6	0.40	7.7
20	1.80	34.6	0.50	9.6
21	1.90	36.5	0.50	9.6
22	1.60	30.8	0.50	9.6
23	1.50	28.8	0.50	9.6
24	1.40	26.9	0.40	7.7
25	1.40	26.9	0.40	7.7
26	1.60	30.8	0.50	9.6
27	1.50	28.8	0.35	6.7
28	1.40	26.9	0.30	5.8
29	1.35	26.0	0.30	5.8
30	1.50	28.8	0.40	7.7
	Average	30.6	Average	8.2
	Standard Deviation	3.6	Standard Deviation	1.3

fas1-2				
No	Measured Conidia Length (cm)	Actual Conidia length (µm)	Measured Conidia	Conidia width (µm)
1	1.90	36.5	0.35	6.7
2	1.60	30.8	0.40	7.7
3	1.30	25.0	0.40	7.7
4	1.60	30.8	0.40	7.7
5	1.60	30.8	0.35	6.7
6	1.60	30.8	0.45	8.7
7	1.40	26.9	0.50	9.6
8	1.65	31.7	0.40	7.7
9	1.80	34.6	0.40	7.7
10	1.80	34.6	0.50	9.6
11	1.80	34.6	0.40	7.7
12	1.80	34.6	0.40	7.7
13	1.70	32.7	0.40	7.7
14	1.50	28.8	0.40	7.7
15	1.60	30.8	0.50	9.6
16	1.50	28.8	0.40	7.7
17	1.40	26.9	0.40	7.7
18	1.50	28.8	0.40	7.7
19	1.30	25.0	0.50	9.6
20	1.80	34.6	0.40	7.7
21	1.80	34.6	0.50	9.6
22	1.80	34.6	0.55	10.6
23	1.70	32.7	0.55	10.6
24	1.70	32.7	0.40	7.7
25	1.65	31.7	0.50	9.6
26	1.60	30.8	0.40	7.7
27	1.50	28.8	0.40	7.7
28	1.40	26.9	0.40	7.7
29	1.50	28.8	0.40	7.7
30	1.60	30.8	0.50	9.6
	Average	31.0	Average	8.3
	Standard Deviation	3.1	Standard Deviation	1.1

Table S7. (A) Conidia length and conidia width measurement of wildtype, *Δfas1-1* and *Δfas1-2* mutants of 10 days under on prune and oat agar plates at temperature 26 ° C. Three set of biological replicate spores filtered through 3 layers of lens paper and counted with a were re-suspended to a final concentration of 5 x 10^4 spores per milliliter. (B) P-value of the measurement of length and width of conidia of wildtype, *Δfas1-1* and *Δfas1-2* mutants of three biological replicates was determined by calculating the significant value using two way anova with replication.

A

Strain	Conidia length (µm)	Conidia width (µm)
**Wild type**	32.2 ± 4.7	8.1 ± 0.9
fas1-1	30.6 ± 3.6	8.2 ± 1.3
fas1-2	31.0 ± 3.1	8.3 ± 1.1

B

Media	Significant	P value	Mean of wild	Mean of fas1-1	Difference	SE of difference	t ratio	df	Adjusted P value
Conidia length	No	0.1188	32.28	30.57	1.707	1.078	1.583	58.00	0.2375
Conidia width	No	0.5279	8.020	8.203	-0.1833	0.2887	0.6351	58.00	>0.9999

Media	Significant	P value	Mean of wild	Mean of fas1-1	Difference	SE of difference	t ratio	df	Adjusted P value
Conidia length	No	0.1820	32.28	30.90	1.380	1.022	1.351	58.00	0.3640
Conidia width	No	0.2495	8.020	8.323	-0.3033	0.2608	1.163	58.00	0.4990

Table S8. DNA extraction buffer, CTAB (Hexacetyltrimethylammonium bromide) buffer recipe

CTAB (Hexacetyltrimethylammonium bromide) extraction buffer

NaCl	10.23 g
EDTA	0.57 g
CTAB	3. 0 g
Tris	3.03 g

dH_2O was added to a final volume of 250 mL and autoclave at 121°C for 20 minutes

Table S9. Sodium dodecyl sulfate polyacrylamide gel electrophoresis (SDS-PAGE) sample buffer recipe.

2x Sample buffer (20 mL)

0.5 M Tris-HCl (pH 6.8)	5 mL
Glycerol	4 mL
SDS	1g
B-mecaptoethanol	0.5 mL
Bromophenol blue	0.005 g

Distilled water was added up to 20 mL, stored in -20 ℃
